# Crystal structures of three newly synthesized flavanone hydrazones

**DOI:** 10.1107/S2056989023001184

**Published:** 2023-02-28

**Authors:** Hemant P. Yennawar, Anna Sigmon, Eleanora Margulis

**Affiliations:** a Department of Biochemistry and Molecular Biology Pennsylvania State University, University Park, PA 16802, USA; b Pennsylvania State University, Brandywine Campus, 25 Yearsley Mill Road, Media, Pa 19063, USA; Texas A & M University, USA

**Keywords:** crystal structure, naringenin, hydrazone, flavanone, flavonoid

## Abstract

The crystal structures of racemic mixtures of three new flavanone-hydrazones in the centrosymmetric space group (*P*  ), are reported. The structures of (±,*E*)-*N*′-[5,7-dihy­droxy-2-(4-hy­droxy­phen­yl)chroman-4-yl­idene]-2-(naphthalen-1-yl)acetohydrazide ethyl acetate monosolvate, C_27_H_22_N_2_O_5_·C_4_H_8_O_2_, and of (±,*E*)-*N*′-[5,7-dihy­droxy-2-(4-hy­droxy­phen­yl)chroman-4-yl­idene]-4-hy­droxy­benzo­hydra­zide ethanol monosolvate, C_22_H_18_N_2_O_6_·C_2_H_5_OH, both exhibit an intra­molecular O—H⋯N and multiple inter­molecular O—H⋯O and C—H⋯O-type hydro­gen bonds. The third structure, that of (±,*E*)-*N*′-(6-methoxy-2-phenyl­chroman-4-yl­idene)-2-(naphthalen-1-yl­oxy)acetohydrazide, C_28_H_24_N_2_O_4_, has only one inter­molecular N—H⋯O-type hydrogen bond. In each of the three cases, the crystal packings are stabilized by π–π stacking inter­actions between various aromatic components of symmetry-related mol­ecules. The chiral carbon atom of the substituted chromane ring system in each case is puckered away from rest of the ring system.

## Abstract 2

The crystal structures of racemic mixtures of three new flavanone-hydrazones are reported: (±,*E*)-*N*′-[5,7-dihy­droxy-2-(4-hy­droxy­phen­yl)chroman-4-yl­idene]-2-(naphthalen-1-yl)acetohydrazide ethyl acetate monosolvate, (±,*E*)-*N*′-[5,7-dihy­droxy-2-(4-hy­droxy­phen­yl)chroman-4-yl­idene]-4-hy­droxy­benzohydrazide ethanol monosolvate and (±,*E*)-*N*′-(6-meth­oxy-2-phenyl­chroman-4-yl­idene)-2-(naphthalen-1-yl­oxy)acetohydrazide. All three hydrazones are in the *E* isomeric form and exhibit a pucker at the chiral carbon atom. The naringenin-derived hydrazones both show intra­molecular hydrogen bonding between the hydrazone nitro­gen atom and a hy­droxy group on the chromane ring.

## 1. Chemical context

Flavonoids encompass a family of organic, naturally occurring polyphenolic compounds with a general structure consisting of a 15-carbon skeleton containing two phenyl rings and a heterocyclic ring. Flavonoids include various subcategories – chalcones, flavones, flavanones, flavanols, isoflavones, anthocyanins – all of which have demonstrated differential health benefits such as anti-­oxidative, anti-inflammatory, anti-mutagenic, and anti-carcinogenic properties (Panche *et al.*, 2016 ▸). As a result of their biologically privileged scaffold, flavonoids and their synthetic derivatives are of significant inter­est to the medicinal chemistry community as potential treatments of disease. We recently reported the first crystal structure of a hydrazone derivative of naringenin, (*R*/*S*,*E*)-2-(4-hy­droxy­phen­yl)-4-(2-phenyl­hydrazineyl­idene)chromane-5,7-diol, a biologically active compound that has been reported to induce apoptosis in human cervical cancer cells (Yennawar & Sigmon, 2022 ▸; Kim *et al.*, 2012 ▸). To further explore the medicinal potential of this class of compounds, three new flavonoid hydrazone compounds have been synthesized and structurally characterized. The three novel compounds are: (±,*E*)-*N*′-[5,7-dihy­droxy-2-(4-hy­droxy­phen­yl)chroman-4-yl­idene]-2-(naphthalen-1-yl)acetohydrazide ethyl acetate monosolvate (**I**), (±,*E*)-*N*′-(5,7-dihy­droxy-2-(4-hy­droxy­phen­yl)chroman-4-yl­idene)-4-hy­droxy­benzohydrazide ethanol monosolvate **(II**) and, (±,*E*)-*N*′-(6-meth­oxy-2-phenyl­chroman-4-yl­idene)-2-(naphthalen-1-yl­oxy)-acetohydrazide (**III**). 
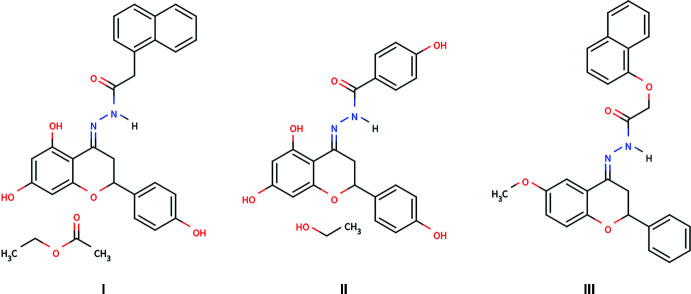


## 2. Structural commentary

**Figure 1:**
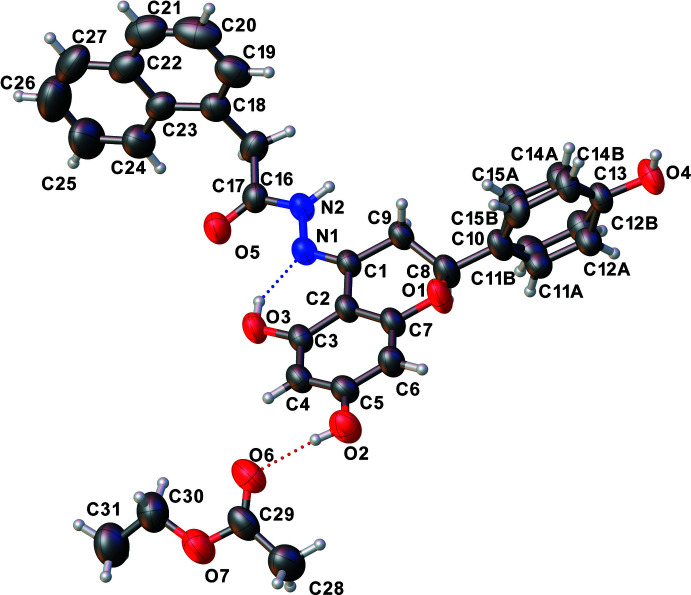
Asymmetric unit of **I** with displacement ellipsoids drawn at the 50% probability level.

Each of the three title compounds (Figs. 1 ▸, 2 ▸ and 3 ▸) has a carbon–nitro­gen double bond [N1=C1: 1.291 (3), 1.294 (4) and 1.284 (5) Å] and all are in the *E* isomeric form. The pyran ring of the chromane ring system in each structure has an envelope pucker with values of the puckering amplitude *Q* of 0.423 (3), 0.397 (6), 0.331 (5) Å, and of θ = 57.9 (4), 53.9 (6), 58.1 (7)°, respectively. The chiral carbon (C8) in each case is displaced between 0.454 and 0.580 Å from the chromane ring planes. The puckering is similar to that seen in the previously reported structure (Yennawar & Sigmon, 2022 ▸).

In compound **I**, the disordered fractions (65/35%) of the 4-hy­droxy­phenyl ring makes dihedral angles of 77.128 (5) and 83.872 (5)°, respectively, with the chromane ring system. An intra­mol­ecular O—H⋯N hydrogen bond exists between one of the hy­droxy groups on the chromane ring and the nitro­gen of the hydrazone group [O—H⋯N = 2.527 (2) Å, 147°]. Another hy­droxy group on the chromane ring participates in a hydrogen bond with the carbonyl group of the solvent ethyl acetate mol­ecule [O2—H2⋯O6 = 2.720 (3) Å, 173°]. The naphthalene ring system is close to perpendicular to the chromane ring system [dihedral angle 77.692 (5)°].

In **II**, the 4-hy­droxy­phenyl ring of the hydrazone moiety is coplanar with the chromane ring [dihedral angle of 2.485 (3)° with the chromane ring system] whereas the other hy­droxy­phenyl ring is almost perpendicular [75.449 (5)°] to the chromane ring system. The chiral carbon of chromane ring (C8_1) and the methyl carbon (C2_1) of the solvent mol­ecule show positional disorder. An *intra*­mol­ecular O—H⋯N hydrogen bond exists between one of the hy­droxy groups on the chromane ring and the nitro­gen of the hydrazone group [O3—H3⋯N1 = 2.542 (3) Å, 147°].

In **III**, the phenyl ring makes a dihedral angle of 86.17 (1)° with the chromane ring system, while the naphthalene ring system is perpendicular to the chromane ring system [dihedral angle = 89.65 (1)°].

## 3. Supra­molecular features

**Figure 4:**
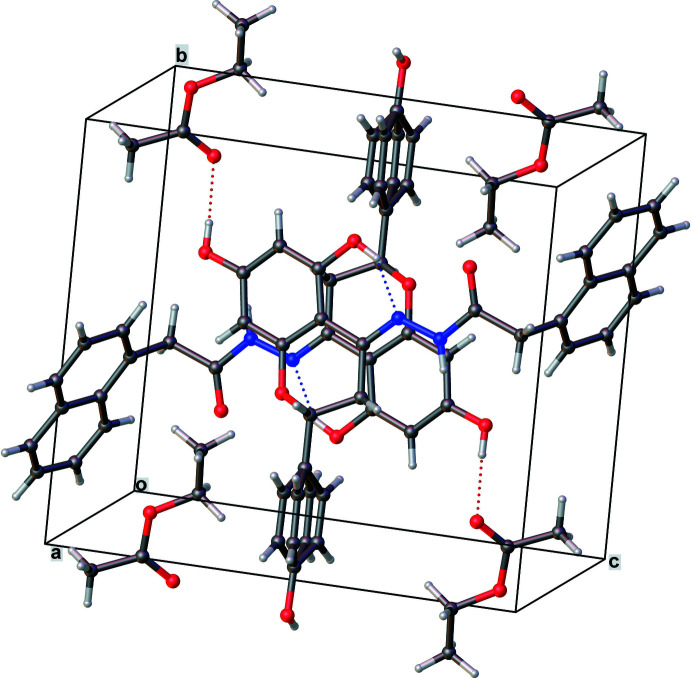
Crystal packing diagram for **I** showing intra­molecular O—H⋯N and inter­molecular O—H⋯O and C—H⋯O hydrogen bonds, as well as extensive π–π stacking inter­actions between aromatic groups.

The extended packing of both **I** and **II** (Figs. 4 ▸ and 5 ▸) exhibit inter­molecular O—H⋯O and C—H⋯O-type inter­actions. Additionally **II** has N—H⋯O-type inter­actions (Tables 1 ▸ and 2 ▸). Both these packings have solvent mol­ecules, namely ethyl acetate and ethanol, respectively, which inter­act with the parent mol­ecules *via* O—H⋯O-type hydrogen bonds. In **I**, π–π inter­actions between the chromane rings of symmetry-related neighbors in the [101] direction are observed. The hy­droxy­phenyl rings also show similar stacking with their symmetry-related counterparts along the [10 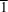 ] direction. Partial stacking (π–π) inter­actions [centroid–centroid distance = 4.51 (1) Å] are observed between the chromane unit and the 4-hydroxyphenyl ring of the hydrazone moiety.

**Figure 6:**
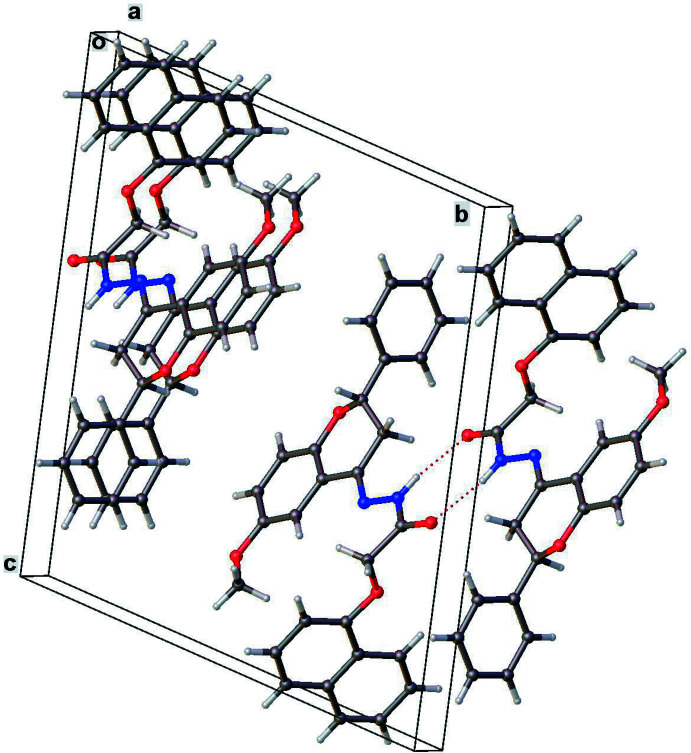
Crystal packing diagram for **III** showing inter­molecular parallel N—H⋯O hydrogen bond pairs, and the π–π stacking inter­actions.

In **III** (Fig. 6 ▸) the hydrogen-bond inter­action is limited to one N—H⋯O-type hydrogen bond between the hydrazone group and carbonyl oxygen of a symmetry-related neighbor [N2—H2⋯O3 = 2.922 (4) Å, 167°], in a parallel, mutual give-and-take fashion (Table 3 ▸). Parallel, partial stacking between symmetry-related naphthalene rings [centroid–centroid distance = 3.790 (2) Å], and also between the chromane ring system and the hydrazone group of neighboring mol­ecules [centroid–centroid distance = 3.730 (3) Å] further stabilizes the packing.

## 4. Database survey

A structure search was performed in Scifinder and Reaxys, and no identical structures were found. A text search (‘flavanone’ and ‘chroman-4-yl­idene’ and ‘chromane-5,7-diol’ and ‘benzo­pyran-4-yl­idene’ and ‘chromen-4-yl­idene’) was performed in the CCDC’s free Access Structures online database (Groom *et al.*, 2016; accessed January, 2023 ▸). Six structures were found of hydrazone derivatives of flavanones, including our previously reported naringenin derivative (Yennawar & Sigmon, 2022 ▸). No crystal structures were found of flavanone hydrazones containing a naphthalene moiety. Examples of other flavanone hydrazones for which crystal data have been reported include acyl hydrazone derivatives of 2-phen­ylchroman-4-one and hesperetin. In particular, crystal structures for 2′-[2-(4-fluoro­phen­yl)­chroman-4-yl­idene]iso­nico­tinohydrazide (Nie *et al.*, 2006 ▸) and *N*-{(±)-[5,7-di­hy­droxy-2-(3-hy­droxy-4-meth­oxy-phen­yl)­chroman-4-yl­idene]am­ino}­benzamide (Lodyga-Chruscinska *et al.*, 2015 ▸) have been reported.

## 5. Synthesis and crystallization

For the preparation of **I**, naringenin (653 mg, 2.4 mmol) and 2-(naphthalen-1-yl)acetohydrazide (501 mg, 2.5 mmol) were dissolved in ethanol (10 mL). Acetic acid (2.4 mmol, 137 µL) was added and the resultant solution was heated at reflux for 21 h. The precipitate was isolated *via* vacuum filtration and recrystallized from ethyl acetate *via* slow evaporation at room temperature to furnish clear, plate-shaped crystals suitable for X-ray analysis.

For the preparation of **II**, naringenin (3.000 g, 11.02 mmol) and 4-hy­droxy­benzohydrazine (2.011 g, 13.22 mmol) were dissolved in ethanol (20 mL). Acetic acid (17.5 mmol, 1.0 mL) was added and the resultant solution was heated at reflux for 48 h. The precipitate was isolated *via* filtration and recrystallized from ethanol *via* slow evaporation at room temperature to furnish transparent yellow, plate-shaped crystals suitable for X-ray analysis.

For the preparation of **III**, 6-meth­oxy­flavanone (381 mg, 1.5 mmol), 2-(naphthalen-1-yl)acetohydrazide (356.8 mg, 1.1 eq, 1.65 mmol), and *p*-toluene­sulfonic acid (29 mg, 0.10 eq, 0.15 mmol) were dissolved in toluene (15mL). The resultant mixture was heated at reflux for 12 h with a Dean–Stark apparatus. The solvent was removed and the crude product was purified on an automated flash chromatography system using a normal phase silica gel column with a gradient of hexa­ne:ethyl acetate (70:30 to 0:100). Recrystallization of the purified compound from ethanol *via* slow evaporation at room temperature furnished yellow, needle-shaped crystals suitable for X-ray analysis.

## 6. Refinement

Crystal data, data collection and structure refinement details for all three structures are summarized in Table 4 ▸. The hydrogen atoms were placed in their geometrically calculated positions and their coordinates refined using the riding model with parent-atom—H lengths of 0.93 Å (CH), 0.98 Å (chiral-CH), 0.96 Å (CH_3_), 0.97 Å (CH_2_), 0.86 Å (NH) or 0.82 Å (OH). Isotropic displacement parameters for these atoms were set to 1.2 (CH, NH) or 1.5 (CH_3_, OH) times *U*_eq_ of the parent atom. In **II**, the positional disorder of the chiral carbon (C8) and phen­oxy ring atoms (C10 through C15) refined to a percentage population ratio of 66/34, and that of the solvent (ethanol) mol­ecule to 57/43, necessitating the use of a total of 136 restraints. The idealized Me of the ethanol mol­ecule were refined as rotating group(s): C2_3 and C2_4 (H2*A*_3 through H2*C*_4).

## Supplementary Material

Crystal structure: contains datablock(s) I, II, III, global. DOI: 10.1107/S2056989023001184/jy2026sup1.cif

Structure factors: contains datablock(s) I. DOI: 10.1107/S2056989023001184/jy2026Isup2.hkl

Supporting information file. DOI: 10.1107/S2056989023001184/jy2026Isup5.mol

Structure factors: contains datablock(s) II. DOI: 10.1107/S2056989023001184/jy2026IIsup3.hkl

Supporting information file. DOI: 10.1107/S2056989023001184/jy2026IIsup6.mol

Structure factors: contains datablock(s) III. DOI: 10.1107/S2056989023001184/jy2026IIIsup4.hkl

Supporting information file. DOI: 10.1107/S2056989023001184/jy2026IIIsup7.mol

Supporting information file. DOI: 10.1107/S2056989023001184/jy2026Isup8.cml

Supporting information file. DOI: 10.1107/S2056989023001184/jy2026IIsup9.cml

Supporting information file. DOI: 10.1107/S2056989023001184/jy2026IIIsup10.cml

hkl file for data_I. DOI: 10.1107/S2056989023001184/jy2026sup11.txt

hkl file for data_II. DOI: 10.1107/S2056989023001184/jy2026sup12.txt

hkl file for data_III. DOI: 10.1107/S2056989023001184/jy2026sup13.txt

RES files for data_block I. DOI: 10.1107/S2056989023001184/jy2026sup14.txt

RES file for data_block II. DOI: 10.1107/S2056989023001184/jy2026sup15.txt

RES file for data_block III. DOI: 10.1107/S2056989023001184/jy2026sup16.txt

matched hkl file for data_III. DOI: 10.1107/S2056989023001184/jy2026sup17.txt

matched hkl file for data_II. DOI: 10.1107/S2056989023001184/jy2026sup18.txt

matched hkl file for data_I. DOI: 10.1107/S2056989023001184/jy2026sup19.txt

SHELX data. DOI: 10.1107/S2056989023001184/jy2026sup20.zip

SHELX data. DOI: 10.1107/S2056989023001184/jy2026sup21.zip

Structure 1 res file. DOI: 10.1107/S2056989023001184/jy2026sup22.txt

Structure I hkl file. DOI: 10.1107/S2056989023001184/jy2026sup23.txt

Structure II res file. DOI: 10.1107/S2056989023001184/jy2026sup24.txt

Structure II hkl file. DOI: 10.1107/S2056989023001184/jy2026sup25.txt

Structure III res file. DOI: 10.1107/S2056989023001184/jy2026sup26.txt

Structure III hkl file. DOI: 10.1107/S2056989023001184/jy2026sup27.txt

CCDC references: 2240752, 2240751, 2240750

Additional supporting information:  crystallographic information; 3D view; checkCIF report

## Figures and Tables

**Figure 2:**
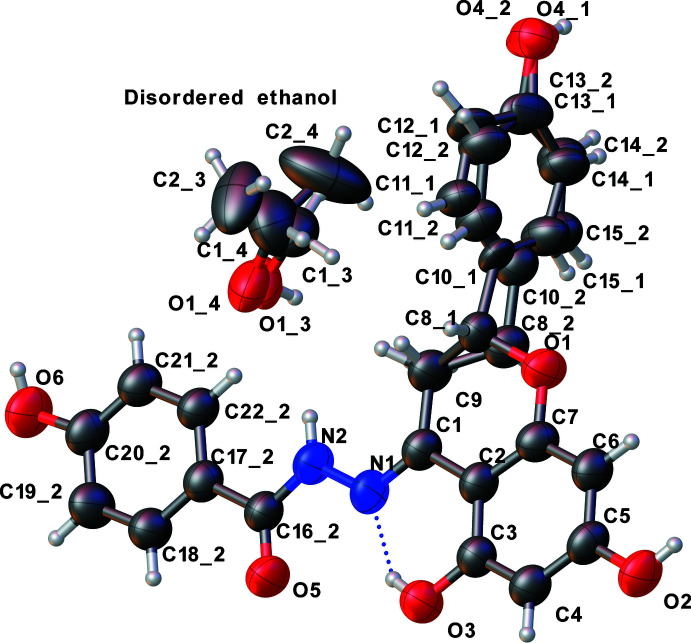
Asymmetric unit of **II** with displacement ellipsoids drawn at the 50% probability level.

**Figure 3:**
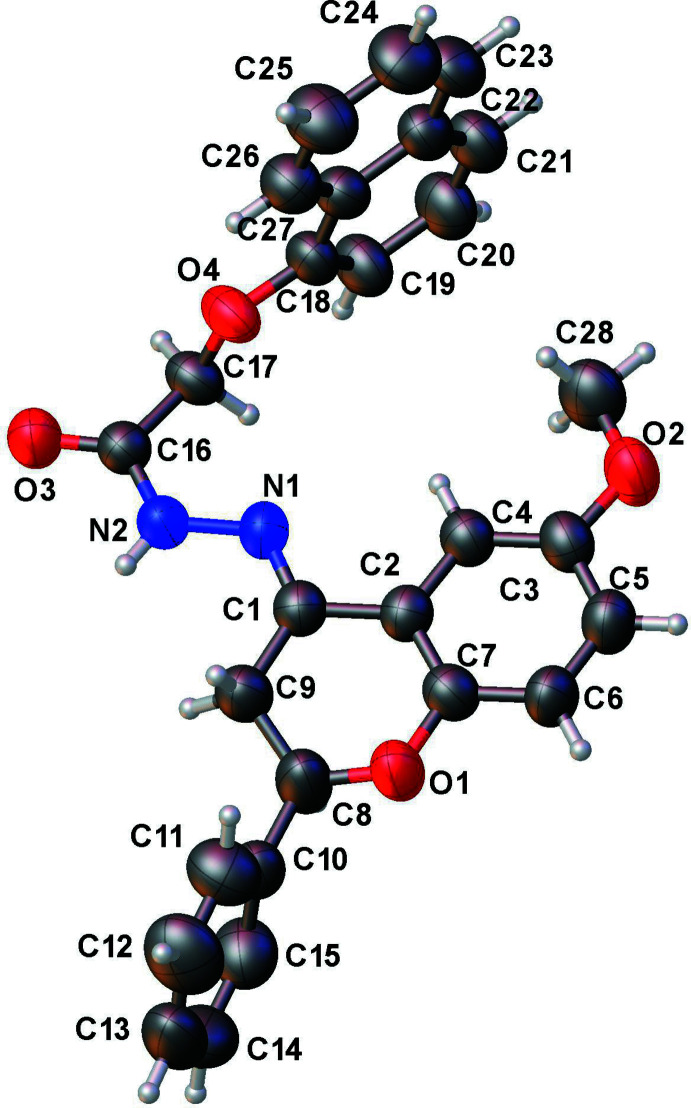
Asymmetric unit of **III** with displacement ellipsoids drawn at the 50% probability level.

**Figure 5:**
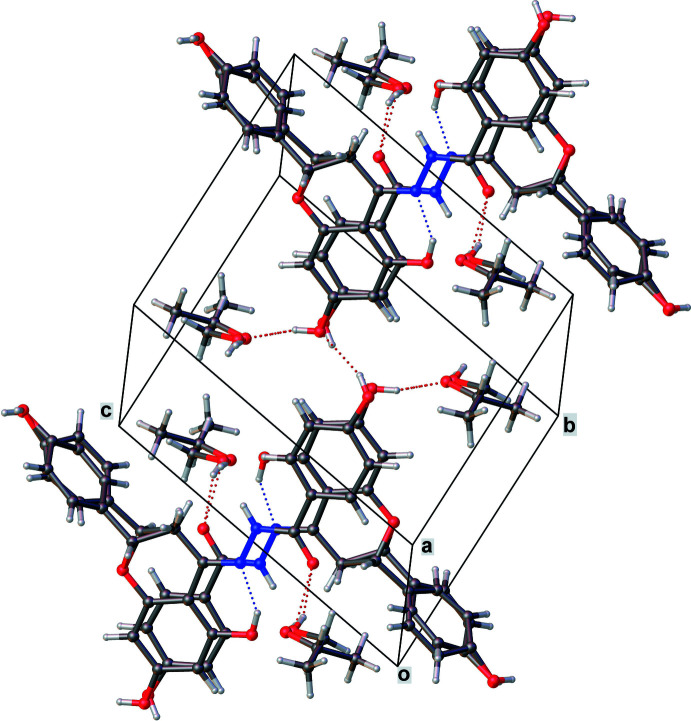
Crystal packing diagram for **II** showing intra­molecular O—H⋯N, inter­molecular O—H⋯O, N—H⋯O, C—H⋯O hydrogen bonds, as well as π–π stacking inter­actions.

**Table 1:** Hydrogen-bond geometry (Å, °) for **I** [Chem scheme1]

*D*—H⋯*A*	*D*—H	H⋯*A*	*D*⋯*A*	*D*—H⋯*A*
O3—H3⋯N1	0.82	1.80	2.527 (2)	147
O2—H2⋯O6	0.82	1.91	2.720 (3)	173
O4—H4⋯O3^i^	0.82	1.93	2.730 (3)	166
C9—H9*B*⋯O4^ii^	0.97	2.57	3.461 (3)	153

Symmetry codes: (i) 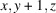 ; (ii) 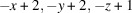 .

**Table 2:** Hydrogen-bond geometry (Å, °) for **II** [Chem scheme1]

*D*—H⋯*A*	*D*—H	H⋯*A*	*D*⋯*A*	*D*—H⋯*A*
O3—H3⋯N1	0.82	1.82	2.542 (3)	147
O6—H6⋯O2^i^	0.82	2.12	2.739 (4)	132
O4_1—H4_1⋯O3^ii^	0.82	1.87	2.52 (3)	136
O4_2—H4_2⋯O3^ii^	0.82	1.96	2.728 (15)	156
O1_3—H1_3⋯O5^iii^	0.82	1.92	2.62 (2)	144
O1_4—H1_4⋯O5^iii^	0.82	2.28	2.877 (17)	130

Symmetry codes: (i) 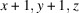 ; (ii) 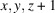 ; (iii) 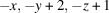 .

**Table 3:** Hydrogen-bond geometry (Å, °) for **III** [Chem scheme1]

*D*—H⋯*A*	*D*—H	H⋯*A*	*D*⋯*A*	*D*—H⋯*A*
N2—H2⋯O3^i^	0.86	2.08	2.922 (4)	167

Symmetry code: (i) 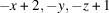 .

**Table 4:** Experimental details

	**I**	**II**	**III**
Crystal data			
Chemical formula	C_27_H_22_N_2_O_5_·C_4_H_8_O_2_	C_22_H_18_N_2_O_6_·C_2_H_6_O	C_28_H_24_N_2_O_4_
*M*_r_	542.57	452.45	452.49
Crystal system, space group	Triclinic, *P* 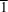	Triclinic, *P* 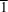	Triclinic, *P* 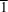
Temperature (K)	293	293	293
*a*, *b*, *c* (Å)	9.2210 (5), 12.1902 (8), 13.4982 (7)	10.0964 (9), 10.1570 (8), 12.3628 (10)	5.0681 (6), 13.4993 (15), 17.1144 (18)
α, β, γ (°)	94.413 (5), 95.172 (4), 111.561 (5)	84.557 (7), 68.169 (8), 82.529 (7)	74.392 (9), 86.34 (1), 88.416 (10)
*V* (Å^3^)	1395.40 (15)	1165.39 (18)	1125.4 (2)
*Z*	2	2	2
Radiation type	Cu *K*α	Cu *K*α	Cu *K*α
μ (mm^−1^)	0.76	0.80	0.73
Crystal size (mm)	0.12 × 0.1 × 0.02	0.18 × 0.16 × 0.04	0.17 × 0.04 × 0.03

Data collection			
Diffractometer	ROD, Synergy Custom system, HyPix-Arc 150	ROD, Synergy Custom system, HyPix-Arc 150	ROD, Synergy Custom system, HyPix-Arc 150
Absorption correction	Multi-scan (*CrysAlis PRO*; Rigaku OD, 2022 ▸)	Multi-scan (*CrysAlis PRO*; Rigaku OD, 2022 ▸)	Multi-scan (*CrysAlis PRO*; Rigaku OD, 2022 ▸)
*T*_min_, *T*_max_	0.912, 1.000	0.660, 1.000	0.889, 1.000
No. of measured, independent and observed [*I* > 2σ(*I*)] reflections	15709, 5498, 3477	12522, 4585, 2642	11899, 4404, 1823
*R*_int_	0.034	0.045	0.051
(sin θ/λ)_max_ (Å^−1^)	0.629	0.637	0.631

Refinement			
*R*[*F* ^2^ > 2σ(*F* ^2^)], *wR*(*F* ^2^), *S*	0.062, 0.212, 1.09	0.086, 0.301, 1.07	0.077, 0.285, 0.99
No. of reflections	5498	4585	4404
No. of parameters	404	405	309
No. of restraints	60	136	6
H-atom treatment	H-atom parameters constrained	H-atom parameters constrained	H-atom parameters constrained
Δρ_max_, Δρ_min_ (e Å^−3^)	0.50, −0.25	0.45, −0.27	0.31, −0.24

Computer programs: *CrysAlis PRO* (Rigaku OD, 2022 ▸), *OLEX2.solve* (Bourhis *et al.*, 2015 ▸), *SHELXL2018/3* (Sheldrick, 2015 ▸), and *OLEX2* (Dolomanov *et al.*, 2009 ▸).

## supplementary crystallographic information

### (±,E)-N'-[5,7-Dihydroxy-2-(4-hydroxyphenyl)chroman-4-ylidene]-2-(naphthalen-1-yl)acetohydrazide ethyl acetate monosolvate (I) Crystal data

**Table:**

C_27_H_22_N_2_O_5_·C_4_H_8_O_2_	*Z* = 2
*M**_r_* = 542.57	*F*(000) = 572
Triclinic, *P*1	*D*_x_ = 1.291 Mg m^−^^3^
*a* = 9.2210 (5) Å	Cu *K*α radiation, λ = 1.54184 Å
*b* = 12.1902 (8) Å	Cell parameters from 7321 reflections
*c* = 13.4982 (7) Å	θ = 3.3–75.2°
α = 94.413 (5)°	µ = 0.76 mm^−^^1^
β = 95.172 (4)°	*T* = 293 K
γ = 111.561 (5)°	Plate, clear colourless
*V* = 1395.40 (15) Å^3^	0.12 × 0.1 × 0.02 mm

### (±,E)-N'-[5,7-Dihydroxy-2-(4-hydroxyphenyl)chroman-4-ylidene]-2-(naphthalen-1-yl)acetohydrazide ethyl acetate monosolvate (I) Data collection

**Table:**

ROD, Synergy Custom system, HyPix-Arc 150 diffractometer	5498 independent reflections
Radiation source: Rotating-anode X-ray tube, Rigaku (Cu) X-ray Source	3477 reflections with *I* > 2σ(*I*)
Mirror monochromator	*R*_int_ = 0.034
Detector resolution: 10.0000 pixels mm^-1^	θ_max_ = 75.9°, θ_min_ = 3.3°
ω scans	*h* = −11→11
Absorption correction: multi-scan (CrysAlisPro; Rigaku OD, 2022)	*k* = −15→14
*T*_min_ = 0.912, *T*_max_ = 1.000	*l* = −16→12
15709 measured reflections	

### (±,E)-N'-[5,7-Dihydroxy-2-(4-hydroxyphenyl)chroman-4-ylidene]-2-(naphthalen-1-yl)acetohydrazide ethyl acetate monosolvate (I) Refinement

**Table:**

Refinement on *F*^2^	Hydrogen site location: inferred from neighbouring sites
Least-squares matrix: full	H-atom parameters constrained
*R*[*F*^2^ > 2σ(*F*^2^)] = 0.062	*w* = 1/[σ^2^(*F*_o_^2^) + (0.1145*P*)^2^ + 0.0876*P*] where *P* = (*F*_o_^2^ + 2*F*_c_^2^)/3
*wR*(*F*^2^) = 0.212	(Δ/σ)_max_ < 0.001
*S* = 1.09	Δρ_max_ = 0.50 e Å^−^^3^
5498 reflections	Δρ_min_ = −0.25 e Å^−^^3^
404 parameters	Extinction correction: SHELXL-2018/3 (Sheldrick 2015), Fc^*^=kFc[1+0.001xFc^2^λ^3^/sin(2θ)]^-1/4^
60 restraints	Extinction coefficient: 0.0043 (8)
Primary atom site location: iterative	

### (±,E)-N'-[5,7-Dihydroxy-2-(4-hydroxyphenyl)chroman-4-ylidene]-2-(naphthalen-1-yl)acetohydrazide ethyl acetate monosolvate (I) Special details

**Table:**

Geometry. All esds (except the esd in the dihedral angle between two l.s. planes) are estimated using the full covariance matrix. The cell esds are taken into account individually in the estimation of esds in distances, angles and torsion angles; correlations between esds in cell parameters are only used when they are defined by crystal symmetry. An approximate (isotropic) treatment of cell esds is used for estimating esds involving l.s. planes.

### (±,E)-N'-[5,7-Dihydroxy-2-(4-hydroxyphenyl)chroman-4-ylidene]-2-(naphthalen-1-yl)acetohydrazide ethyl acetate monosolvate (I) Fractional atomic coordinates and isotropic or equivalent isotropic displacement parameters (Å^2^)

**Table:**

	*x*	*y*	*z*	*U*_iso_*/*U*_eq_	Occ. (<1)
O1	0.6529 (2)	0.67088 (14)	0.64906 (14)	0.0691 (5)	
O2	0.3755 (3)	0.32732 (16)	0.78886 (14)	0.0732 (6)	
H2	0.357223	0.255910	0.784880	0.110*	
O3	0.6861 (2)	0.29835 (14)	0.53395 (12)	0.0587 (5)	
H3	0.734294	0.337833	0.492432	0.088*	
O4	0.8931 (2)	1.22094 (14)	0.63511 (15)	0.0699 (5)	
H4	0.820055	1.232642	0.604745	0.105*	
O5	0.9167 (2)	0.35560 (15)	0.32740 (13)	0.0676 (5)	
N1	0.8079 (2)	0.48109 (16)	0.44746 (14)	0.0523 (5)	
N2	0.8883 (2)	0.52798 (17)	0.37020 (14)	0.0559 (5)	
H2A	0.907953	0.600522	0.359940	0.067*	
C1	0.7675 (3)	0.54747 (19)	0.50946 (17)	0.0504 (5)	
C2	0.6743 (3)	0.48816 (19)	0.58533 (16)	0.0495 (5)	
C3	0.6336 (3)	0.36660 (19)	0.59418 (17)	0.0497 (5)	
C4	0.5359 (3)	0.3116 (2)	0.66263 (17)	0.0556 (6)	
H4A	0.510446	0.231204	0.667796	0.067*	
C5	0.4763 (3)	0.3773 (2)	0.72333 (18)	0.0561 (6)	
C6	0.5177 (3)	0.4979 (2)	0.71828 (19)	0.0616 (7)	
H6	0.478794	0.541832	0.760104	0.074*	
C7	0.6165 (3)	0.5524 (2)	0.65115 (18)	0.0547 (6)	
C8	0.7937 (4)	0.7331 (2)	0.6102 (2)	0.0692 (8)	
H8	0.879916	0.729332	0.656079	0.083*	
C9	0.8033 (3)	0.6775 (2)	0.50939 (17)	0.0562 (6)	
H9A	0.729095	0.689158	0.459701	0.067*	
H9B	0.908012	0.716126	0.491192	0.067*	
C10	0.8150 (4)	0.8617 (2)	0.6144 (2)	0.0618 (7)	
C11A	0.941 (4)	0.942 (2)	0.6829 (18)	0.057 (4)	0.29 (3)
H11A	1.009448	0.917739	0.721640	0.068*	0.29 (3)
C11B	0.9501 (17)	0.9468 (11)	0.6569 (10)	0.075 (3)	0.71 (3)
H11B	1.029305	0.923893	0.684204	0.090*	0.71 (3)
C12A	0.957 (3)	1.061 (2)	0.6892 (16)	0.054 (4)	0.29 (3)
H12A	1.032911	1.115939	0.737333	0.065*	0.29 (3)
C12B	0.9790 (14)	1.0664 (10)	0.6628 (10)	0.073 (3)	0.71 (3)
H12B	1.075991	1.122273	0.691794	0.088*	0.71 (3)
C13	0.8629 (3)	1.1015 (2)	0.62542 (18)	0.0552 (6)	
C14A	0.754 (3)	1.017 (2)	0.561 (2)	0.063 (6)	0.29 (3)
H14A	0.716016	1.036696	0.501339	0.076*	0.29 (3)
C14B	0.7203 (11)	1.0200 (8)	0.5770 (8)	0.0591 (17)	0.71 (3)
H14B	0.637252	1.042150	0.555969	0.071*	0.71 (3)
C15A	0.696 (4)	0.897 (3)	0.583 (2)	0.060 (5)	0.29 (3)
H15A	0.590208	0.848653	0.577167	0.072*	0.29 (3)
C15B	0.7098 (16)	0.8998 (12)	0.5617 (11)	0.074 (3)	0.71 (3)
H15B	0.630181	0.845746	0.515181	0.089*	0.71 (3)
C16	0.9357 (3)	0.4563 (2)	0.31054 (17)	0.0557 (6)	
C17	1.0111 (3)	0.5114 (2)	0.22271 (18)	0.0657 (7)	
H17A	1.113605	0.506219	0.223075	0.079*	
H17B	1.025891	0.594748	0.228703	0.079*	
C18	0.9101 (3)	0.4491 (2)	0.12479 (18)	0.0599 (6)	
C19	0.8009 (4)	0.4899 (3)	0.0847 (2)	0.0792 (9)	
H19	0.787197	0.554213	0.118333	0.095*	
C20	0.7074 (4)	0.4338 (4)	−0.0093 (3)	0.0987 (12)	
H20	0.632133	0.460805	−0.036553	0.118*	
C21	0.7296 (5)	0.3416 (4)	−0.0578 (3)	0.0965 (11)	
H21	0.669012	0.306306	−0.118958	0.116*	
C22	0.8380 (4)	0.2977 (3)	−0.0206 (2)	0.0786 (9)	
C23	0.9276 (3)	0.3499 (2)	0.07340 (18)	0.0619 (7)	
C24	1.0342 (4)	0.3000 (3)	0.1086 (2)	0.0826 (9)	
H24	1.094272	0.331436	0.170610	0.099*	
C25	1.0540 (6)	0.2075 (4)	0.0561 (3)	0.1191 (15)	
H25	1.125586	0.176640	0.082417	0.143*	
C26	0.9669 (7)	0.1600 (4)	−0.0366 (4)	0.1339 (18)	
H26	0.982094	0.098063	−0.073114	0.161*	
C27	0.8617 (5)	0.2017 (4)	−0.0744 (3)	0.1051 (13)	
H27	0.803198	0.167824	−0.136491	0.126*	
O6	0.3415 (3)	0.09561 (19)	0.78449 (17)	0.0915 (7)	
O7	0.3890 (3)	−0.04819 (19)	0.85469 (17)	0.0935 (7)	
C28	0.3659 (5)	0.1020 (3)	0.9623 (3)	0.1020 (12)	
H28A	0.358033	0.178276	0.961757	0.153*	
H28B	0.462489	0.110491	1.001444	0.153*	
H28C	0.278770	0.049573	0.991100	0.153*	
C29	0.3634 (4)	0.0516 (2)	0.8580 (2)	0.0724 (8)	
C30	0.3861 (6)	−0.1082 (3)	0.7563 (3)	0.1097 (13)	
H30A	0.449610	−0.051887	0.715369	0.132*	
H30B	0.279194	−0.143361	0.722555	0.132*	
C31	0.4493 (5)	−0.2009 (4)	0.7716 (3)	0.1133 (13)	
H31A	0.432660	−0.250460	0.709538	0.170*	
H31B	0.396736	−0.248231	0.820839	0.170*	
H31C	0.559927	−0.164463	0.794426	0.170*	

### (±,E)-N'-[5,7-Dihydroxy-2-(4-hydroxyphenyl)chroman-4-ylidene]-2-(naphthalen-1-yl)acetohydrazide ethyl acetate monosolvate (I) Atomic displacement parameters (Å^2^)

**Table:**

	*U*^11^	*U*^22^	*U*^33^	*U*^12^	*U*^13^	*U*^23^
O1	0.0919 (14)	0.0403 (9)	0.0881 (12)	0.0322 (9)	0.0401 (11)	0.0149 (8)
O2	0.0844 (14)	0.0609 (11)	0.0837 (12)	0.0294 (11)	0.0366 (11)	0.0241 (10)
O3	0.0746 (12)	0.0409 (8)	0.0697 (11)	0.0291 (8)	0.0206 (9)	0.0091 (7)
O4	0.0734 (13)	0.0367 (9)	0.0982 (14)	0.0202 (9)	0.0059 (10)	0.0081 (8)
O5	0.0899 (14)	0.0518 (10)	0.0704 (11)	0.0340 (10)	0.0232 (10)	0.0093 (8)
N1	0.0621 (13)	0.0438 (10)	0.0561 (11)	0.0235 (9)	0.0148 (9)	0.0089 (8)
N2	0.0724 (14)	0.0402 (10)	0.0598 (11)	0.0232 (10)	0.0201 (10)	0.0102 (8)
C1	0.0600 (15)	0.0398 (11)	0.0555 (12)	0.0230 (11)	0.0095 (11)	0.0074 (9)
C2	0.0575 (14)	0.0393 (11)	0.0571 (12)	0.0233 (10)	0.0106 (11)	0.0073 (9)
C3	0.0532 (14)	0.0405 (11)	0.0589 (13)	0.0220 (10)	0.0069 (11)	0.0057 (9)
C4	0.0626 (16)	0.0428 (12)	0.0643 (14)	0.0220 (11)	0.0094 (12)	0.0115 (10)
C5	0.0628 (16)	0.0511 (13)	0.0609 (14)	0.0257 (12)	0.0145 (12)	0.0146 (11)
C6	0.0747 (18)	0.0519 (14)	0.0676 (15)	0.0308 (13)	0.0239 (13)	0.0097 (11)
C7	0.0654 (16)	0.0417 (12)	0.0648 (14)	0.0272 (11)	0.0138 (12)	0.0102 (10)
C8	0.089 (2)	0.0471 (14)	0.0797 (17)	0.0298 (14)	0.0289 (15)	0.0120 (12)
C9	0.0717 (17)	0.0420 (12)	0.0617 (14)	0.0266 (12)	0.0160 (12)	0.0114 (10)
C10	0.0781 (19)	0.0458 (13)	0.0735 (16)	0.0326 (14)	0.0247 (14)	0.0125 (12)
C11A	0.076 (8)	0.048 (7)	0.049 (7)	0.029 (5)	−0.004 (6)	0.003 (5)
C11B	0.085 (4)	0.061 (3)	0.090 (6)	0.040 (3)	0.004 (5)	0.019 (4)
C12A	0.068 (8)	0.047 (6)	0.054 (7)	0.030 (6)	0.001 (6)	0.005 (5)
C12B	0.070 (4)	0.051 (3)	0.096 (6)	0.023 (3)	0.000 (4)	0.007 (4)
C13	0.0627 (16)	0.0356 (11)	0.0691 (15)	0.0194 (11)	0.0127 (12)	0.0069 (10)
C14A	0.063 (9)	0.064 (7)	0.075 (8)	0.040 (6)	0.002 (6)	0.006 (6)
C14B	0.051 (3)	0.043 (2)	0.087 (4)	0.023 (2)	0.011 (3)	0.001 (2)
C15A	0.055 (7)	0.049 (7)	0.062 (8)	0.004 (5)	0.009 (6)	−0.002 (6)
C15B	0.087 (5)	0.045 (3)	0.081 (6)	0.018 (3)	0.002 (4)	−0.003 (3)
C16	0.0619 (16)	0.0452 (12)	0.0579 (13)	0.0180 (11)	0.0104 (11)	0.0015 (10)
C17	0.0754 (19)	0.0553 (15)	0.0600 (15)	0.0158 (13)	0.0196 (13)	0.0017 (11)
C18	0.0638 (16)	0.0591 (15)	0.0588 (14)	0.0209 (13)	0.0206 (12)	0.0158 (11)
C19	0.081 (2)	0.082 (2)	0.087 (2)	0.0371 (17)	0.0281 (18)	0.0308 (16)
C20	0.064 (2)	0.127 (3)	0.108 (3)	0.031 (2)	0.009 (2)	0.055 (2)
C21	0.085 (3)	0.111 (3)	0.075 (2)	0.013 (2)	0.0124 (18)	0.019 (2)
C22	0.078 (2)	0.079 (2)	0.0632 (17)	0.0101 (17)	0.0154 (15)	0.0095 (15)
C23	0.0668 (17)	0.0615 (15)	0.0526 (13)	0.0161 (13)	0.0172 (12)	0.0093 (11)
C24	0.103 (3)	0.084 (2)	0.0773 (18)	0.050 (2)	0.0276 (17)	0.0138 (16)
C25	0.170 (4)	0.117 (3)	0.109 (3)	0.091 (3)	0.051 (3)	0.015 (3)
C26	0.189 (6)	0.103 (3)	0.118 (4)	0.060 (4)	0.056 (4)	−0.006 (3)
C27	0.127 (4)	0.089 (3)	0.071 (2)	0.011 (2)	0.023 (2)	−0.0125 (18)
O6	0.1160 (19)	0.0635 (13)	0.1004 (15)	0.0374 (13)	0.0119 (13)	0.0261 (11)
O7	0.131 (2)	0.0734 (14)	0.0982 (15)	0.0582 (14)	0.0304 (14)	0.0243 (11)
C28	0.134 (3)	0.098 (3)	0.099 (2)	0.064 (3)	0.043 (2)	0.0249 (19)
C29	0.0725 (19)	0.0546 (15)	0.096 (2)	0.0243 (14)	0.0256 (16)	0.0242 (14)
C30	0.150 (4)	0.080 (2)	0.106 (3)	0.053 (3)	0.015 (3)	0.000 (2)
C31	0.131 (4)	0.100 (3)	0.129 (3)	0.065 (3)	0.035 (3)	0.004 (2)

### (±,E)-N'-[5,7-Dihydroxy-2-(4-hydroxyphenyl)chroman-4-ylidene]-2-(naphthalen-1-yl)acetohydrazide ethyl acetate monosolvate (I) Geometric parameters (Å, º)

**Table:**

O1—C7	1.361 (3)	C14A—H14A	0.9300
O1—C8	1.413 (3)	C14A—C15A	1.43 (3)
O2—H2	0.8200	C14B—H14B	0.9300
O2—C5	1.353 (3)	C14B—C15B	1.430 (14)
O3—H3	0.8200	C15A—H15A	0.9300
O3—C3	1.361 (3)	C15B—H15B	0.9300
O4—H4	0.8200	C16—C17	1.506 (3)
O4—C13	1.371 (3)	C17—H17A	0.9700
O5—C16	1.217 (3)	C17—H17B	0.9700
N1—N2	1.372 (3)	C17—C18	1.515 (4)
N1—C1	1.291 (3)	C18—C19	1.367 (4)
N2—H2A	0.8600	C18—C23	1.415 (4)
N2—C16	1.356 (3)	C19—H19	0.9300
C1—C2	1.458 (3)	C19—C20	1.434 (5)
C1—C9	1.496 (3)	C20—H20	0.9300
C2—C3	1.406 (3)	C20—C21	1.346 (5)
C2—C7	1.404 (3)	C21—H21	0.9300
C3—C4	1.384 (3)	C21—C22	1.372 (5)
C4—H4A	0.9300	C22—C23	1.413 (4)
C4—C5	1.385 (3)	C22—C27	1.426 (5)
C5—C6	1.386 (3)	C23—C24	1.400 (4)
C6—H6	0.9300	C24—H24	0.9300
C6—C7	1.374 (3)	C24—C25	1.363 (4)
C8—H8	0.9800	C25—H25	0.9300
C8—C9	1.497 (3)	C25—C26	1.383 (7)
C8—C10	1.502 (3)	C26—H26	0.9300
C9—H9A	0.9700	C26—C27	1.331 (6)
C9—H9B	0.9700	C27—H27	0.9300
C10—C11A	1.41 (3)	O6—C29	1.199 (3)
C10—C11B	1.337 (13)	O7—C29	1.320 (3)
C10—C15A	1.36 (3)	O7—C30	1.459 (4)
C10—C15B	1.386 (13)	C28—H28A	0.9600
C11A—H11A	0.9300	C28—H28B	0.9600
C11A—C12A	1.40 (3)	C28—H28C	0.9600
C11B—H11B	0.9300	C28—C29	1.487 (5)
C11B—C12B	1.377 (14)	C30—H30A	0.9700
C12A—H12A	0.9300	C30—H30B	0.9700
C12A—C13	1.41 (2)	C30—C31	1.469 (5)
C12B—H12B	0.9300	C31—H31A	0.9600
C12B—C13	1.362 (12)	C31—H31B	0.9600
C13—C14A	1.33 (2)	C31—H31C	0.9600
C13—C14B	1.388 (9)		
			
C7—O1—C8	115.96 (18)	C13—C14B—C15B	115.5 (9)
C5—O2—H2	109.5	C15B—C14B—H14B	122.2
C3—O3—H3	109.5	C10—C15A—C14A	112 (2)
C13—O4—H4	109.5	C10—C15A—H15A	124.1
C1—N1—N2	120.20 (19)	C14A—C15A—H15A	124.1
N1—N2—H2A	121.1	C10—C15B—C14B	121.7 (11)
C16—N2—N1	117.90 (19)	C10—C15B—H15B	119.1
C16—N2—H2A	121.1	C14B—C15B—H15B	119.1
N1—C1—C2	116.19 (19)	O5—C16—N2	121.9 (2)
N1—C1—C9	126.6 (2)	O5—C16—C17	123.7 (2)
C2—C1—C9	117.23 (18)	N2—C16—C17	114.5 (2)
C3—C2—C1	122.97 (18)	C16—C17—H17A	109.4
C7—C2—C1	119.7 (2)	C16—C17—H17B	109.4
C7—C2—C3	117.3 (2)	C16—C17—C18	111.0 (2)
O3—C3—C2	121.0 (2)	H17A—C17—H17B	108.0
O3—C3—C4	117.68 (19)	C18—C17—H17A	109.4
C4—C3—C2	121.30 (19)	C18—C17—H17B	109.4
C3—C4—H4A	120.3	C19—C18—C17	119.4 (3)
C3—C4—C5	119.5 (2)	C19—C18—C23	119.2 (3)
C5—C4—H4A	120.3	C23—C18—C17	121.3 (2)
O2—C5—C4	122.1 (2)	C18—C19—H19	120.0
O2—C5—C6	117.4 (2)	C18—C19—C20	120.1 (3)
C4—C5—C6	120.6 (2)	C20—C19—H19	120.0
C5—C6—H6	120.2	C19—C20—H20	120.3
C7—C6—C5	119.7 (2)	C21—C20—C19	119.3 (3)
C7—C6—H6	120.2	C21—C20—H20	120.3
O1—C7—C2	121.2 (2)	C20—C21—H21	118.6
O1—C7—C6	117.2 (2)	C20—C21—C22	122.7 (3)
C6—C7—C2	121.6 (2)	C22—C21—H21	118.6
O1—C8—H8	106.8	C21—C22—C23	118.5 (3)
O1—C8—C9	113.1 (2)	C21—C22—C27	121.6 (3)
O1—C8—C10	108.5 (2)	C23—C22—C27	119.9 (3)
C9—C8—H8	106.8	C22—C23—C18	120.0 (3)
C9—C8—C10	114.5 (2)	C24—C23—C18	123.8 (3)
C10—C8—H8	106.8	C24—C23—C22	116.1 (3)
C1—C9—C8	111.10 (19)	C23—C24—H24	118.5
C1—C9—H9A	109.4	C25—C24—C23	123.0 (3)
C1—C9—H9B	109.4	C25—C24—H24	118.5
C8—C9—H9A	109.4	C24—C25—H25	120.2
C8—C9—H9B	109.4	C24—C25—C26	119.5 (4)
H9A—C9—H9B	108.0	C26—C25—H25	120.2
C11A—C10—C8	116.4 (14)	C25—C26—H26	119.5
C11B—C10—C8	120.6 (7)	C27—C26—C25	120.9 (4)
C11B—C10—C15B	116.0 (9)	C27—C26—H26	119.5
C15A—C10—C8	122.4 (14)	C22—C27—H27	119.7
C15A—C10—C11A	118 (2)	C26—C27—C22	120.6 (4)
C15B—C10—C8	122.7 (6)	C26—C27—H27	119.7
C10—C11A—H11A	121.9	C29—O7—C30	117.7 (3)
C12A—C11A—C10	116 (3)	H28A—C28—H28B	109.5
C12A—C11A—H11A	121.9	H28A—C28—H28C	109.5
C10—C11B—H11B	118.0	H28B—C28—H28C	109.5
C10—C11B—C12B	124.0 (12)	C29—C28—H28A	109.5
C12B—C11B—H11B	118.0	C29—C28—H28B	109.5
C11A—C12A—H12A	118.7	C29—C28—H28C	109.5
C11A—C12A—C13	123 (2)	O6—C29—O7	123.1 (3)
C13—C12A—H12A	118.7	O6—C29—C28	124.7 (3)
C11B—C12B—H12B	120.6	O7—C29—C28	112.2 (3)
C13—C12B—C11B	118.8 (11)	O7—C30—H30A	110.2
C13—C12B—H12B	120.6	O7—C30—H30B	110.2
O4—C13—C12A	117.7 (11)	O7—C30—C31	107.6 (3)
O4—C13—C14B	120.9 (5)	H30A—C30—H30B	108.5
C12B—C13—O4	117.6 (5)	C31—C30—H30A	110.2
C12B—C13—C14B	121.5 (7)	C31—C30—H30B	110.2
C14A—C13—O4	127.3 (11)	C30—C31—H31A	109.5
C14A—C13—C12A	115.0 (16)	C30—C31—H31B	109.5
C13—C14A—H14A	120.1	C30—C31—H31C	109.5
C13—C14A—C15A	120 (2)	H31A—C31—H31B	109.5
C15A—C14A—H14A	120.1	H31A—C31—H31C	109.5
C13—C14B—H14B	122.2	H31B—C31—H31C	109.5
			
O1—C8—C9—C1	−50.2 (3)	C9—C8—C10—C11B	−104.4 (7)
O1—C8—C10—C11A	111.6 (14)	C9—C8—C10—C15A	81.1 (15)
O1—C8—C10—C11B	128.3 (7)	C9—C8—C10—C15B	65.0 (8)
O1—C8—C10—C15A	−46.3 (16)	C10—C8—C9—C1	−175.2 (2)
O1—C8—C10—C15B	−62.4 (8)	C10—C11A—C12A—C13	−5 (3)
O2—C5—C6—C7	−178.3 (2)	C10—C11B—C12B—C13	1.6 (14)
O3—C3—C4—C5	−178.2 (2)	C11A—C10—C15A—C14A	43 (3)
O4—C13—C14A—C15A	−156 (2)	C11A—C12A—C13—O4	−177.2 (19)
O4—C13—C14B—C15B	173.1 (9)	C11A—C12A—C13—C14A	2 (3)
O5—C16—C17—C18	66.2 (4)	C11B—C10—C15B—C14B	−19 (2)
N1—N2—C16—O5	−4.2 (4)	C11B—C12B—C13—O4	178.0 (7)
N1—N2—C16—C17	175.2 (2)	C11B—C12B—C13—C14B	−3.6 (11)
N1—C1—C2—C3	2.0 (4)	C12A—C13—C14A—C15A	25 (3)
N1—C1—C2—C7	−175.6 (2)	C12B—C13—C14B—C15B	−5.3 (12)
N1—C1—C9—C8	−158.5 (3)	C13—C14A—C15A—C10	−48 (4)
N2—N1—C1—C2	176.4 (2)	C13—C14B—C15B—C10	17 (2)
N2—N1—C1—C9	−2.0 (4)	C15A—C10—C11A—C12A	−19 (3)
N2—C16—C17—C18	−113.2 (3)	C15B—C10—C11B—C12B	9.3 (15)
C1—N1—N2—C16	176.4 (2)	C16—C17—C18—C19	91.2 (3)
C1—C2—C3—O3	3.0 (4)	C16—C17—C18—C23	−89.4 (3)
C1—C2—C3—C4	−175.5 (2)	C17—C18—C19—C20	178.3 (3)
C1—C2—C7—O1	−3.5 (4)	C17—C18—C23—C22	−176.4 (2)
C1—C2—C7—C6	174.6 (2)	C17—C18—C23—C24	1.5 (4)
C2—C1—C9—C8	23.1 (3)	C18—C19—C20—C21	−0.7 (5)
C2—C3—C4—C5	0.3 (4)	C18—C23—C24—C25	−177.2 (3)
C3—C2—C7—O1	178.6 (2)	C19—C18—C23—C22	3.0 (4)
C3—C2—C7—C6	−3.2 (4)	C19—C18—C23—C24	−179.1 (3)
C3—C4—C5—O2	177.4 (2)	C19—C20—C21—C22	0.6 (5)
C3—C4—C5—C6	−2.1 (4)	C20—C21—C22—C23	1.3 (5)
C4—C5—C6—C7	1.2 (4)	C20—C21—C22—C27	−178.6 (3)
C5—C6—C7—O1	179.8 (2)	C21—C22—C23—C18	−3.1 (4)
C5—C6—C7—C2	1.5 (4)	C21—C22—C23—C24	178.8 (3)
C7—O1—C8—C9	52.2 (3)	C21—C22—C27—C26	−179.6 (4)
C7—O1—C8—C10	−179.6 (2)	C22—C23—C24—C25	0.8 (5)
C7—C2—C3—O3	−179.3 (2)	C23—C18—C19—C20	−1.1 (4)
C7—C2—C3—C4	2.3 (4)	C23—C22—C27—C26	0.5 (6)
C8—O1—C7—C2	−24.6 (4)	C23—C24—C25—C26	0.5 (6)
C8—O1—C7—C6	157.2 (2)	C24—C25—C26—C27	−1.4 (7)
C8—C10—C11A—C12A	−177.8 (16)	C25—C26—C27—C22	0.9 (7)
C8—C10—C11B—C12B	179.4 (7)	C27—C22—C23—C18	176.8 (3)
C8—C10—C15A—C14A	−159.4 (18)	C27—C22—C23—C24	−1.3 (4)
C8—C10—C15B—C14B	171.4 (10)	C29—O7—C30—C31	168.3 (3)
C9—C1—C2—C3	−179.4 (2)	C30—O7—C29—O6	−1.6 (5)
C9—C1—C2—C7	2.9 (3)	C30—O7—C29—C28	178.6 (3)
C9—C8—C10—C11A	−121.0 (14)		

### (±,E)-N'-[5,7-Dihydroxy-2-(4-hydroxyphenyl)chroman-4-ylidene]-2-(naphthalen-1-yl)acetohydrazide ethyl acetate monosolvate (I) Hydrogen-bond geometry (Å, º)

**Table:**

*D*—H···*A*	*D*—H	H···*A*	*D*···*A*	*D*—H···*A*
O3—H3···N1	0.82	1.80	2.527 (2)	147
O2—H2···O6	0.82	1.91	2.720 (3)	173
O4—H4···O3^i^	0.82	1.93	2.730 (3)	166
C9—H9*B*···O4^ii^	0.97	2.57	3.461 (3)	153

Symmetry codes: (i) *x*, *y*+1, *z*; (ii) −*x*+2, −*y*+2, −*z*+1.

### (±,E)-N'-[5,7-Dihydroxy-2-(4-hydroxyphenyl)chroman-4-ylidene]-4-hydroxybenzohydrazide ethanol monosolvate (II) Crystal data

**Table:**

C_22_H_18_N_2_O_6_·C_2_H_6_O	*Z* = 2
*M**_r_* = 452.45	*F*(000) = 476
Triclinic, *P*1	*D*_x_ = 1.289 Mg m^−^^3^
*a* = 10.0964 (9) Å	Cu *K*α radiation, λ = 1.54184 Å
*b* = 10.1570 (8) Å	Cell parameters from 5399 reflections
*c* = 12.3628 (10) Å	θ = 3.9–76.0°
α = 84.557 (7)°	µ = 0.80 mm^−^^1^
β = 68.169 (8)°	*T* = 293 K
γ = 82.529 (7)°	Plate, yellow
*V* = 1165.39 (18) Å^3^	0.18 × 0.16 × 0.04 mm

### (±,E)-N'-[5,7-Dihydroxy-2-(4-hydroxyphenyl)chroman-4-ylidene]-4-hydroxybenzohydrazide ethanol monosolvate (II) Data collection

**Table:**

ROD, Synergy Custom system, HyPix-Arc 150 diffractometer	4585 independent reflections
Radiation source: Rotating-anode X-ray tube, Rigaku (Cu) X-ray Source	2642 reflections with *I* > 2σ(*I*)
Mirror monochromator	*R*_int_ = 0.045
Detector resolution: 10.0000 pixels mm^-1^	θ_max_ = 79.0°, θ_min_ = 3.9°
ω scans	*h* = −12→11
Absorption correction: multi-scan (CrysAlisPro; Rigaku OD, 2022)	*k* = −11→12
*T*_min_ = 0.660, *T*_max_ = 1.000	*l* = −15→14
12522 measured reflections	

### (±,E)-N'-[5,7-Dihydroxy-2-(4-hydroxyphenyl)chroman-4-ylidene]-4-hydroxybenzohydrazide ethanol monosolvate (II) Refinement

**Table:**

Refinement on *F*^2^	Hydrogen site location: mixed
Least-squares matrix: full	H-atom parameters constrained
*R*[*F*^2^ > 2σ(*F*^2^)] = 0.086	*w* = 1/[σ^2^(*F*_o_^2^) + (0.1916*P*)^2^] where *P* = (*F*_o_^2^ + 2*F*_c_^2^)/3
*wR*(*F*^2^) = 0.301	(Δ/σ)_max_ < 0.001
*S* = 1.07	Δρ_max_ = 0.45 e Å^−^^3^
4585 reflections	Δρ_min_ = −0.27 e Å^−^^3^
405 parameters	Extinction correction: SHELXL-2018/3 (Sheldrick 2015), Fc^*^=kFc[1+0.001xFc^2^λ^3^/sin(2θ)]^-1/4^
136 restraints	Extinction coefficient: 0.015 (3)

### (±,E)-N'-[5,7-Dihydroxy-2-(4-hydroxyphenyl)chroman-4-ylidene]-4-hydroxybenzohydrazide ethanol monosolvate (II) Special details

**Table:**

Geometry. All esds (except the esd in the dihedral angle between two l.s. planes) are estimated using the full covariance matrix. The cell esds are taken into account individually in the estimation of esds in distances, angles and torsion angles; correlations between esds in cell parameters are only used when they are defined by crystal symmetry. An approximate (isotropic) treatment of cell esds is used for estimating esds involving l.s. planes.

### (±,E)-N'-[5,7-Dihydroxy-2-(4-hydroxyphenyl)chroman-4-ylidene]-4-hydroxybenzohydrazide ethanol monosolvate (II) Fractional atomic coordinates and isotropic or equivalent isotropic displacement parameters (Å^2^)

**Table:**

	*x*	*y*	*z*	*U*_iso_*/*U*_eq_	Occ. (<1)
O1	−0.1351 (2)	0.6849 (2)	0.79739 (19)	0.0754 (7)	
O2	−0.3070 (2)	0.4492 (2)	0.5747 (2)	0.0785 (7)	
H2	−0.342557	0.411258	0.638824	0.118*	
O3	0.0348 (2)	0.7463 (2)	0.37844 (18)	0.0724 (6)	
H3	0.085010	0.796329	0.390234	0.109*	
O5	0.3098 (3)	0.9705 (2)	0.3030 (2)	0.0837 (7)	
O6	0.6730 (3)	1.3952 (3)	0.3686 (2)	0.0925 (8)	
H6	0.640291	1.442748	0.424580	0.139*	
N1	0.1291 (3)	0.8729 (2)	0.4979 (2)	0.0644 (7)	
N2	0.2153 (3)	0.9675 (2)	0.4986 (2)	0.0654 (7)	
H2A	0.211270	0.997485	0.562629	0.079*	
C1	0.0487 (3)	0.8194 (3)	0.5956 (3)	0.0607 (7)	
C2	−0.0431 (3)	0.7220 (3)	0.5882 (3)	0.0610 (7)	
C3	−0.0481 (3)	0.6899 (3)	0.4820 (3)	0.0609 (8)	
C4	−0.1388 (3)	0.6004 (3)	0.4786 (3)	0.0639 (8)	
H4	−0.144130	0.582551	0.407948	0.077*	
C5	−0.2211 (3)	0.5383 (3)	0.5811 (3)	0.0654 (8)	
C6	−0.2188 (3)	0.5665 (3)	0.6873 (3)	0.0682 (8)	
H6A	−0.275641	0.524598	0.756009	0.082*	
C7	−0.1302 (3)	0.6585 (3)	0.6892 (3)	0.0643 (8)	
C9	0.0375 (4)	0.8466 (4)	0.7136 (3)	0.0741 (9)	
H9AA	0.045689	0.940341	0.715850	0.089*	0.340 (11)
H9AB	0.117221	0.796585	0.729797	0.089*	0.340 (11)
H9BC	0.130296	0.866285	0.710629	0.089*	0.660 (11)
H9BD	−0.029867	0.924754	0.739493	0.089*	0.660 (11)
O4_1	−0.106 (5)	0.864 (3)	1.260 (2)	0.073 (5)	0.340 (11)
H4_1	−0.030662	0.825179	1.262161	0.110*	0.340 (11)
C8_1	−0.0995 (14)	0.8119 (11)	0.8069 (7)	0.066 (3)	0.340 (11)
H8_1	−0.176753	0.877540	0.800365	0.079*	0.340 (11)
C10_1	−0.097 (2)	0.8167 (15)	0.9282 (10)	0.061 (3)	0.340 (11)
C11_1	−0.175 (2)	0.9296 (17)	0.9914 (13)	0.070 (3)	0.340 (11)
H11_1	−0.225729	0.993774	0.958569	0.084*	0.340 (11)
C12_1	−0.176 (3)	0.943 (2)	1.1033 (17)	0.077 (4)	0.340 (11)
H12_1	−0.209873	1.023414	1.139562	0.092*	0.340 (11)
C13_1	−0.1256 (16)	0.8330 (19)	1.1593 (13)	0.056 (3)	0.340 (11)
C14_1	−0.039 (2)	0.730 (2)	1.0937 (16)	0.080 (5)	0.340 (11)
H14_1	0.009772	0.665175	1.126783	0.096*	0.340 (11)
C15_1	−0.026 (2)	0.7250 (18)	0.9820 (14)	0.076 (4)	0.340 (11)
H15_1	0.034812	0.655947	0.939016	0.092*	0.340 (11)
O4_2	−0.135 (2)	0.8700 (16)	1.2634 (11)	0.077 (3)	0.660 (11)
H4_2	−0.096223	0.813666	1.297414	0.115*	0.660 (11)
C8_2	−0.0097 (7)	0.7352 (7)	0.7987 (4)	0.0706 (19)	0.660 (11)
H8_2	0.068019	0.662710	0.777363	0.085*	0.660 (11)
C10_2	−0.0381 (10)	0.7645 (10)	0.9230 (6)	0.071 (2)	0.660 (11)
C11_2	−0.1351 (10)	0.8742 (10)	0.9700 (7)	0.076 (2)	0.660 (11)
H11_2	−0.178298	0.925612	0.923456	0.092*	0.660 (11)
C12_2	−0.1690 (17)	0.9089 (12)	1.0835 (10)	0.083 (3)	0.660 (11)
H12_2	−0.243121	0.974346	1.116940	0.099*	0.660 (11)
C13_2	−0.0882 (10)	0.8423 (11)	1.1457 (8)	0.064 (2)	0.660 (11)
C14_2	0.0007 (13)	0.7302 (12)	1.1015 (9)	0.078 (2)	0.660 (11)
H14_2	0.043577	0.677733	1.147780	0.094*	0.660 (11)
C15_2	0.0274 (9)	0.6940 (9)	0.9912 (7)	0.078 (2)	0.660 (11)
H15_2	0.091550	0.619884	0.962366	0.093*	0.660 (11)
C16_2	0.3063 (3)	1.0107 (3)	0.3936 (3)	0.0636 (8)	
C17_2	0.4023 (3)	1.1118 (3)	0.3940 (3)	0.0608 (7)	
C18_2	0.4883 (3)	1.1626 (3)	0.2872 (3)	0.0723 (9)	
H18_2	0.485608	1.132852	0.219239	0.087*	
C19_2	0.5787 (4)	1.2575 (4)	0.2797 (3)	0.0819 (10)	
H19_2	0.636572	1.291323	0.207324	0.098*	
C20_2	0.5820 (3)	1.3016 (3)	0.3814 (3)	0.0707 (9)	
C21_2	0.4985 (3)	1.2513 (3)	0.4867 (3)	0.0693 (8)	
H21_2	0.501184	1.281219	0.554621	0.083*	
C22_2	0.4093 (3)	1.1559 (3)	0.4939 (3)	0.0659 (8)	
H22_2	0.353493	1.121001	0.566623	0.079*	
O1_3	−0.388 (2)	1.274 (2)	0.7634 (16)	0.091 (4)	0.433 (11)
H1_3	−0.340646	1.201827	0.758789	0.136*	0.433 (11)
C1_3	−0.5178 (19)	1.2716 (16)	0.8636 (15)	0.098 (4)	0.433 (11)
H1A_3	−0.494641	1.243379	0.932237	0.117*	0.433 (11)
H1B_3	−0.575177	1.207245	0.853981	0.117*	0.433 (11)
C2_3	−0.6057 (13)	1.4090 (11)	0.8819 (13)	0.143 (6)	0.433 (11)
H2A_3	−0.596826	1.450423	0.806772	0.214*	0.433 (11)
H2B_3	−0.577666	1.466963	0.924742	0.214*	0.433 (11)
H2C_3	−0.703526	1.391903	0.924062	0.214*	0.433 (11)
O1_4	−0.412 (2)	1.3022 (18)	0.7513 (12)	0.106 (4)	0.567 (11)
H1_4	−0.350775	1.242391	0.755089	0.159*	0.567 (11)
C1_4	−0.527 (2)	1.313 (2)	0.8644 (15)	0.163 (8)	0.567 (11)
H1A_4	−0.609437	1.275514	0.862817	0.196*	0.567 (11)
H1B_4	−0.554448	1.406173	0.881159	0.196*	0.567 (11)
C2_4	−0.4798 (13)	1.2400 (19)	0.9615 (9)	0.206 (8)	0.567 (11)
H2A_4	−0.447910	1.148451	0.944346	0.310*	0.567 (11)
H2B_4	−0.556780	1.244951	1.036036	0.310*	0.567 (11)
H2C_4	−0.401730	1.283391	0.963206	0.310*	0.567 (11)

### (±,E)-N'-[5,7-Dihydroxy-2-(4-hydroxyphenyl)chroman-4-ylidene]-4-hydroxybenzohydrazide ethanol monosolvate (II) Atomic displacement parameters (Å^2^)

**Table:**

	*U*^11^	*U*^22^	*U*^33^	*U*^12^	*U*^13^	*U*^23^
O1	0.0832 (14)	0.0856 (15)	0.0645 (14)	−0.0407 (12)	−0.0238 (10)	−0.0078 (11)
O2	0.0804 (14)	0.0765 (14)	0.0888 (16)	−0.0392 (11)	−0.0314 (13)	−0.0090 (12)
O3	0.0789 (14)	0.0811 (15)	0.0653 (13)	−0.0354 (11)	−0.0278 (10)	0.0010 (11)
O5	0.1085 (17)	0.0820 (15)	0.0691 (15)	−0.0476 (13)	−0.0290 (12)	−0.0070 (11)
O6	0.0878 (16)	0.0978 (19)	0.099 (2)	−0.0569 (14)	−0.0259 (13)	−0.0063 (14)
N1	0.0650 (14)	0.0629 (15)	0.0726 (17)	−0.0284 (11)	−0.0269 (12)	0.0000 (12)
N2	0.0682 (15)	0.0660 (15)	0.0703 (16)	−0.0289 (12)	−0.0270 (12)	−0.0056 (12)
C1	0.0607 (16)	0.0604 (17)	0.0678 (18)	−0.0188 (13)	−0.0266 (13)	−0.0051 (13)
C2	0.0598 (16)	0.0607 (17)	0.0676 (19)	−0.0216 (13)	−0.0239 (13)	−0.0037 (14)
C3	0.0626 (16)	0.0604 (17)	0.0646 (18)	−0.0175 (13)	−0.0252 (13)	−0.0030 (13)
C4	0.0621 (16)	0.0664 (18)	0.0709 (19)	−0.0189 (14)	−0.0282 (14)	−0.0068 (15)
C5	0.0598 (16)	0.0637 (18)	0.080 (2)	−0.0207 (13)	−0.0266 (14)	−0.0121 (15)
C6	0.0642 (17)	0.0693 (19)	0.072 (2)	−0.0276 (14)	−0.0191 (14)	−0.0037 (15)
C7	0.0594 (16)	0.0702 (19)	0.0664 (19)	−0.0203 (13)	−0.0202 (13)	−0.0109 (14)
C9	0.079 (2)	0.081 (2)	0.072 (2)	−0.0358 (16)	−0.0279 (16)	−0.0060 (16)
O4_1	0.073 (11)	0.091 (9)	0.065 (7)	−0.011 (6)	−0.029 (6)	−0.029 (6)
C8_1	0.078 (7)	0.060 (6)	0.066 (5)	−0.025 (5)	−0.029 (5)	−0.002 (4)
C10_1	0.093 (11)	0.047 (8)	0.057 (6)	−0.028 (6)	−0.032 (7)	−0.012 (4)
C11_1	0.088 (10)	0.068 (9)	0.060 (7)	−0.012 (6)	−0.032 (6)	−0.012 (5)
C12_1	0.100 (10)	0.090 (9)	0.062 (8)	−0.035 (8)	−0.043 (8)	−0.017 (6)
C13_1	0.040 (7)	0.090 (6)	0.041 (5)	−0.050 (5)	−0.004 (4)	0.001 (4)
C14_1	0.096 (14)	0.094 (8)	0.066 (7)	−0.026 (8)	−0.039 (7)	−0.016 (5)
C15_1	0.094 (12)	0.080 (9)	0.068 (7)	−0.002 (7)	−0.044 (8)	−0.014 (6)
O4_2	0.086 (9)	0.086 (4)	0.061 (3)	−0.022 (4)	−0.027 (3)	−0.003 (3)
C8_2	0.067 (3)	0.078 (4)	0.074 (3)	−0.024 (3)	−0.026 (2)	−0.011 (2)
C10_2	0.076 (5)	0.071 (6)	0.073 (4)	−0.019 (3)	−0.028 (3)	−0.009 (3)
C11_2	0.089 (6)	0.074 (7)	0.075 (5)	−0.015 (4)	−0.037 (5)	−0.012 (4)
C12_2	0.090 (4)	0.096 (6)	0.074 (5)	−0.020 (5)	−0.037 (4)	−0.020 (4)
C13_2	0.047 (4)	0.083 (4)	0.062 (4)	−0.039 (3)	−0.009 (3)	−0.008 (3)
C14_2	0.080 (6)	0.091 (4)	0.072 (4)	−0.024 (4)	−0.030 (3)	−0.015 (3)
C15_2	0.078 (5)	0.085 (5)	0.075 (4)	−0.013 (3)	−0.030 (3)	−0.014 (3)
C16_2	0.0712 (18)	0.0588 (17)	0.0642 (19)	−0.0208 (14)	−0.0236 (14)	−0.0042 (14)
C17_2	0.0573 (15)	0.0597 (17)	0.0670 (19)	−0.0153 (13)	−0.0208 (13)	−0.0053 (13)
C18_2	0.0748 (19)	0.077 (2)	0.067 (2)	−0.0291 (16)	−0.0208 (15)	−0.0048 (15)
C19_2	0.076 (2)	0.096 (2)	0.074 (2)	−0.0429 (18)	−0.0173 (16)	−0.0016 (18)
C20_2	0.0616 (17)	0.073 (2)	0.084 (2)	−0.0257 (14)	−0.0270 (15)	−0.0075 (16)
C21_2	0.0640 (17)	0.073 (2)	0.075 (2)	−0.0218 (15)	−0.0238 (15)	−0.0100 (16)
C22_2	0.0654 (17)	0.0676 (18)	0.0684 (19)	−0.0223 (14)	−0.0228 (14)	−0.0071 (14)
O1_3	0.096 (7)	0.060 (8)	0.086 (6)	−0.019 (4)	0.001 (5)	0.009 (4)
C1_3	0.100 (8)	0.083 (8)	0.091 (9)	−0.023 (6)	−0.006 (5)	−0.012 (6)
C2_3	0.100 (8)	0.080 (7)	0.189 (14)	0.002 (6)	0.017 (7)	−0.022 (8)
O1_4	0.134 (8)	0.069 (8)	0.102 (5)	−0.032 (5)	−0.019 (5)	−0.009 (5)
C1_4	0.179 (15)	0.154 (18)	0.132 (10)	−0.079 (13)	−0.014 (7)	0.018 (10)
C2_4	0.174 (11)	0.36 (2)	0.077 (6)	−0.065 (12)	−0.031 (6)	0.000 (9)

### (±,E)-N'-[5,7-Dihydroxy-2-(4-hydroxyphenyl)chroman-4-ylidene]-4-hydroxybenzohydrazide ethanol monosolvate (II) Geometric parameters (Å, º)

**Table:**

O1—C7	1.370 (4)	C15_1—H15_1	0.9300
O1—C8_1	1.410 (9)	O4_2—H4_2	0.8200
O1—C8_2	1.433 (5)	O4_2—C13_2	1.397 (10)
O2—H2	0.8200	C8_2—H8_2	0.9800
O2—C5	1.359 (3)	C8_2—C10_2	1.506 (8)
O3—H3	0.8200	C10_2—C11_2	1.398 (10)
O3—C3	1.363 (3)	C10_2—C15_2	1.356 (9)
O5—C16_2	1.215 (3)	C11_2—H11_2	0.9300
O6—H6	0.8200	C11_2—C12_2	1.383 (9)
O6—C20_2	1.366 (3)	C12_2—H12_2	0.9300
N1—N2	1.380 (3)	C12_2—C13_2	1.393 (10)
N1—C1	1.294 (4)	C13_2—C14_2	1.375 (9)
N2—H2A	0.8600	C14_2—H14_2	0.9300
N2—C16_2	1.354 (4)	C14_2—C15_2	1.365 (9)
C1—C2	1.471 (3)	C15_2—H15_2	0.9300
C1—C9	1.471 (4)	C16_2—C17_2	1.502 (4)
C2—C3	1.403 (4)	C17_2—C18_2	1.378 (4)
C2—C7	1.388 (4)	C17_2—C22_2	1.382 (4)
C3—C4	1.385 (4)	C18_2—H18_2	0.9300
C4—H4	0.9300	C18_2—C19_2	1.386 (4)
C4—C5	1.377 (4)	C19_2—H19_2	0.9300
C5—C6	1.378 (4)	C19_2—C20_2	1.387 (5)
C6—H6A	0.9300	C20_2—C21_2	1.355 (5)
C6—C7	1.383 (4)	C21_2—H21_2	0.9300
C9—H9AA	0.9700	C21_2—C22_2	1.382 (4)
C9—H9AB	0.9700	C22_2—H22_2	0.9300
C9—H9BC	0.9700	O1_3—H1_3	0.8200
C9—H9BD	0.9700	O1_3—C1_3	1.431 (11)
C9—C8_1	1.494 (11)	C1_3—H1A_3	0.9700
C9—C8_2	1.469 (7)	C1_3—H1B_3	0.9700
O4_1—H4_1	0.8200	C1_3—C2_3	1.541 (12)
O4_1—C13_1	1.405 (15)	C2_3—H2A_3	0.9601
C8_1—H8_1	0.9800	C2_3—H2B_3	0.9599
C8_1—C10_1	1.515 (11)	C2_3—H2C_3	0.9600
C10_1—C11_1	1.424 (14)	O1_4—H1_4	0.8200
C10_1—C15_1	1.375 (14)	O1_4—C1_4	1.448 (12)
C11_1—H11_1	0.9300	C1_4—H1A_4	0.9700
C11_1—C12_1	1.399 (13)	C1_4—H1B_4	0.9700
C12_1—H12_1	0.9300	C1_4—C2_4	1.542 (14)
C12_1—C13_1	1.401 (14)	C2_4—H2A_4	0.9600
C13_1—C14_1	1.379 (14)	C2_4—H2B_4	0.9601
C14_1—H14_1	0.9300	C2_4—H2C_4	0.9600
C14_1—C15_1	1.341 (14)		
			
C7—O1—C8_1	115.5 (4)	O1—C8_2—C10_2	107.3 (4)
C7—O1—C8_2	115.1 (3)	C9—C8_2—H8_2	107.0
C5—O2—H2	109.5	C9—C8_2—C10_2	114.6 (5)
C3—O3—H3	109.5	C10_2—C8_2—H8_2	107.0
C20_2—O6—H6	109.5	C11_2—C10_2—C8_2	117.9 (8)
C1—N1—N2	119.6 (3)	C15_2—C10_2—C8_2	124.3 (8)
N1—N2—H2A	121.7	C15_2—C10_2—C11_2	117.8 (6)
C16_2—N2—N1	116.7 (3)	C10_2—C11_2—H11_2	118.9
C16_2—N2—H2A	121.7	C12_2—C11_2—C10_2	122.3 (7)
N1—C1—C2	116.6 (3)	C12_2—C11_2—H11_2	118.9
N1—C1—C9	127.1 (3)	C11_2—C12_2—H12_2	121.2
C9—C1—C2	116.3 (3)	C11_2—C12_2—C13_2	117.6 (9)
C3—C2—C1	122.7 (3)	C13_2—C12_2—H12_2	121.2
C7—C2—C1	119.9 (3)	C12_2—C13_2—O4_2	116.0 (9)
C7—C2—C3	117.4 (3)	C14_2—C13_2—O4_2	122.3 (9)
O3—C3—C2	121.4 (2)	C14_2—C13_2—C12_2	118.9 (8)
O3—C3—C4	117.4 (3)	C13_2—C14_2—H14_2	119.3
C4—C3—C2	121.2 (3)	C15_2—C14_2—C13_2	121.5 (8)
C3—C4—H4	120.4	C15_2—C14_2—H14_2	119.3
C5—C4—C3	119.1 (3)	C10_2—C15_2—C14_2	121.0 (8)
C5—C4—H4	120.4	C10_2—C15_2—H15_2	119.5
O2—C5—C4	117.7 (3)	C14_2—C15_2—H15_2	119.5
O2—C5—C6	120.7 (3)	O5—C16_2—N2	121.7 (3)
C4—C5—C6	121.5 (3)	O5—C16_2—C17_2	121.4 (3)
C5—C6—H6A	120.7	N2—C16_2—C17_2	116.9 (3)
C5—C6—C7	118.5 (3)	C18_2—C17_2—C16_2	117.1 (3)
C7—C6—H6A	120.7	C18_2—C17_2—C22_2	118.7 (3)
O1—C7—C2	121.9 (2)	C22_2—C17_2—C16_2	124.2 (3)
O1—C7—C6	115.9 (3)	C17_2—C18_2—H18_2	119.6
C6—C7—C2	122.2 (3)	C17_2—C18_2—C19_2	120.9 (3)
C1—C9—H9AA	108.8	C19_2—C18_2—H18_2	119.6
C1—C9—H9AB	108.8	C18_2—C19_2—H19_2	120.4
C1—C9—H9BC	109.0	C18_2—C19_2—C20_2	119.2 (3)
C1—C9—H9BD	109.0	C20_2—C19_2—H19_2	120.4
C1—C9—C8_1	113.6 (4)	O6—C20_2—C19_2	116.6 (3)
H9AA—C9—H9AB	107.7	C21_2—C20_2—O6	123.2 (3)
H9BC—C9—H9BD	107.8	C21_2—C20_2—C19_2	120.2 (3)
C8_1—C9—H9AA	108.8	C20_2—C21_2—H21_2	119.8
C8_1—C9—H9AB	108.8	C20_2—C21_2—C22_2	120.4 (3)
C8_2—C9—C1	113.0 (3)	C22_2—C21_2—H21_2	119.8
C8_2—C9—H9BC	109.0	C17_2—C22_2—H22_2	119.7
C8_2—C9—H9BD	109.0	C21_2—C22_2—C17_2	120.5 (3)
C13_1—O4_1—H4_1	109.4	C21_2—C22_2—H22_2	119.7
O1—C8_1—C9	113.3 (8)	C1_3—O1_3—H1_3	109.5
O1—C8_1—H8_1	107.8	O1_3—C1_3—H1A_3	109.3
O1—C8_1—C10_1	107.5 (7)	O1_3—C1_3—H1B_3	109.3
C9—C8_1—H8_1	107.8	O1_3—C1_3—C2_3	111.6 (12)
C9—C8_1—C10_1	112.3 (8)	H1A_3—C1_3—H1B_3	108.0
C10_1—C8_1—H8_1	107.8	C2_3—C1_3—H1A_3	109.3
C11_1—C10_1—C8_1	115.7 (13)	C2_3—C1_3—H1B_3	109.3
C15_1—C10_1—C8_1	127.0 (13)	C1_3—C2_3—H2A_3	108.3
C15_1—C10_1—C11_1	117.3 (10)	C1_3—C2_3—H2B_3	114.2
C10_1—C11_1—H11_1	120.3	C1_3—C2_3—H2C_3	105.8
C12_1—C11_1—C10_1	119.5 (12)	H2A_3—C2_3—H2B_3	109.5
C12_1—C11_1—H11_1	120.3	H2A_3—C2_3—H2C_3	109.5
C11_1—C12_1—H12_1	120.8	H2B_3—C2_3—H2C_3	109.5
C11_1—C12_1—C13_1	118.4 (13)	C1_4—O1_4—H1_4	109.5
C13_1—C12_1—H12_1	120.8	O1_4—C1_4—H1A_4	109.4
C12_1—C13_1—O4_1	113.3 (16)	O1_4—C1_4—H1B_4	109.4
C14_1—C13_1—O4_1	119.9 (16)	O1_4—C1_4—C2_4	111.3 (13)
C14_1—C13_1—C12_1	119.2 (13)	H1A_4—C1_4—H1B_4	108.0
C13_1—C14_1—H14_1	120.0	C2_4—C1_4—H1A_4	109.4
C15_1—C14_1—C13_1	119.9 (15)	C2_4—C1_4—H1B_4	109.4
C15_1—C14_1—H14_1	120.0	C1_4—C2_4—H2A_4	110.4
C10_1—C15_1—H15_1	118.4	C1_4—C2_4—H2B_4	111.2
C14_1—C15_1—C10_1	123.1 (13)	C1_4—C2_4—H2C_4	106.7
C14_1—C15_1—H15_1	118.4	H2A_4—C2_4—H2B_4	109.5
C13_2—O4_2—H4_2	109.7	H2A_4—C2_4—H2C_4	109.5
O1—C8_2—C9	113.5 (5)	H2B_4—C2_4—H2C_4	109.5
O1—C8_2—H8_2	107.0		
			
O1—C8_1—C10_1—C11_1	−130.6 (15)	C7—C2—C3—O3	−179.2 (3)
O1—C8_1—C10_1—C15_1	50.2 (19)	C7—C2—C3—C4	1.4 (5)
O1—C8_2—C10_2—C11_2	−72.0 (8)	C9—C1—C2—C3	177.4 (3)
O1—C8_2—C10_2—C15_2	110.1 (9)	C9—C1—C2—C7	−2.1 (4)
O2—C5—C6—C7	−179.6 (3)	C9—C8_1—C10_1—C11_1	104.1 (16)
O3—C3—C4—C5	178.0 (3)	C9—C8_1—C10_1—C15_1	−75.1 (17)
O5—C16_2—C17_2—C18_2	3.7 (5)	C9—C8_2—C10_2—C11_2	55.1 (9)
O5—C16_2—C17_2—C22_2	−176.2 (3)	C9—C8_2—C10_2—C15_2	−122.8 (8)
O6—C20_2—C21_2—C22_2	−179.5 (3)	O4_1—C13_1—C14_1—C15_1	−160 (3)
N1—N2—C16_2—O5	1.6 (5)	C8_1—O1—C7—C2	25.8 (7)
N1—N2—C16_2—C17_2	−178.9 (2)	C8_1—O1—C7—C6	−153.2 (7)
N1—C1—C2—C3	−1.8 (5)	C8_1—C10_1—C11_1—C12_1	−178.8 (19)
N1—C1—C2—C7	178.7 (3)	C8_1—C10_1—C15_1—C14_1	−173.7 (18)
N1—C1—C9—C8_1	157.7 (6)	C10_1—C11_1—C12_1—C13_1	−13 (4)
N1—C1—C9—C8_2	−154.8 (4)	C11_1—C10_1—C15_1—C14_1	7 (3)
N2—N1—C1—C2	178.4 (2)	C11_1—C12_1—C13_1—O4_1	169 (3)
N2—N1—C1—C9	−0.7 (5)	C11_1—C12_1—C13_1—C14_1	19 (4)
N2—C16_2—C17_2—C18_2	−175.8 (3)	C12_1—C13_1—C14_1—C15_1	−12 (3)
N2—C16_2—C17_2—C22_2	4.3 (5)	C13_1—C14_1—C15_1—C10_1	−1 (3)
C1—N1—N2—C16_2	174.2 (3)	C15_1—C10_1—C11_1—C12_1	0 (3)
C1—C2—C3—O3	1.2 (5)	O4_2—C13_2—C14_2—C15_2	169.8 (14)
C1—C2—C3—C4	−178.2 (3)	C8_2—O1—C7—C2	−24.7 (5)
C1—C2—C7—O1	0.9 (5)	C8_2—O1—C7—C6	156.3 (4)
C1—C2—C7—C6	179.8 (3)	C8_2—C10_2—C11_2—C12_2	179.6 (10)
C1—C9—C8_1—O1	47.3 (10)	C8_2—C10_2—C15_2—C14_2	177.1 (9)
C1—C9—C8_1—C10_1	169.4 (8)	C10_2—C11_2—C12_2—C13_2	9.0 (19)
C1—C9—C8_2—O1	−49.8 (6)	C11_2—C10_2—C15_2—C14_2	−0.8 (14)
C1—C9—C8_2—C10_2	−173.6 (5)	C11_2—C12_2—C13_2—O4_2	−173.6 (14)
C2—C1—C9—C8_1	−21.4 (7)	C11_2—C12_2—C13_2—C14_2	−12.4 (19)
C2—C1—C9—C8_2	26.1 (5)	C12_2—C13_2—C14_2—C15_2	9.7 (18)
C2—C3—C4—C5	−2.6 (5)	C13_2—C14_2—C15_2—C10_2	−3.0 (17)
C3—C2—C7—O1	−178.7 (3)	C15_2—C10_2—C11_2—C12_2	−2.4 (15)
C3—C2—C7—C6	0.2 (5)	C16_2—C17_2—C18_2—C19_2	179.2 (3)
C3—C4—C5—O2	−178.8 (3)	C16_2—C17_2—C22_2—C21_2	−178.7 (3)
C3—C4—C5—C6	2.3 (5)	C17_2—C18_2—C19_2—C20_2	−0.1 (6)
C4—C5—C6—C7	−0.7 (5)	C18_2—C17_2—C22_2—C21_2	1.5 (5)
C5—C6—C7—O1	178.4 (3)	C18_2—C19_2—C20_2—O6	−179.9 (3)
C5—C6—C7—C2	−0.5 (5)	C18_2—C19_2—C20_2—C21_2	0.6 (6)
C7—O1—C8_1—C9	−49.7 (10)	C19_2—C20_2—C21_2—C22_2	0.0 (5)
C7—O1—C8_1—C10_1	−174.4 (8)	C20_2—C21_2—C22_2—C17_2	−1.0 (5)
C7—O1—C8_2—C9	49.4 (6)	C22_2—C17_2—C18_2—C19_2	−0.9 (5)
C7—O1—C8_2—C10_2	177.2 (5)		

### (±,E)-N'-[5,7-Dihydroxy-2-(4-hydroxyphenyl)chroman-4-ylidene]-4-hydroxybenzohydrazide ethanol monosolvate (II) Hydrogen-bond geometry (Å, º)

**Table:**

*D*—H···*A*	*D*—H	H···*A*	*D*···*A*	*D*—H···*A*
O3—H3···N1	0.82	1.82	2.542 (3)	147
O6—H6···O2^i^	0.82	2.12	2.739 (4)	132
O4_1—H4_1···O3^ii^	0.82	1.87	2.52 (3)	136
O4_2—H4_2···O3^ii^	0.82	1.96	2.728 (15)	156
O1_3—H1_3···O5^iii^	0.82	1.92	2.62 (2)	144
O1_4—H1_4···O5^iii^	0.82	2.28	2.877 (17)	130

Symmetry codes: (i) *x*+1, *y*+1, *z*; (ii) *x*, *y*, *z*+1; (iii) −*x*, −*y*+2, −*z*+1.

### (±,E)-N'-(6-Methoxy-2-phenylchroman-4-ylidene)-2-(naphthalen-1-yloxy)-acetohydrazide (III) Crystal data

**Table:**

C_28_H_24_N_2_O_4_	*Z* = 2
*M**_r_* = 452.49	*F*(000) = 476
Triclinic, *P*1	*D*_x_ = 1.335 Mg m^−^^3^
*a* = 5.0681 (6) Å	Cu *K*α radiation, λ = 1.54184 Å
*b* = 13.4993 (15) Å	Cell parameters from 3831 reflections
*c* = 17.1144 (18) Å	θ = 3.7–73.1°
α = 74.392 (9)°	µ = 0.73 mm^−^^1^
β = 86.34 (1)°	*T* = 293 K
γ = 88.416 (10)°	Needle, clear yellow
*V* = 1125.4 (2) Å^3^	0.17 × 0.04 × 0.03 mm

### (±,E)-N'-(6-Methoxy-2-phenylchroman-4-ylidene)-2-(naphthalen-1-yloxy)-acetohydrazide (III) Data collection

**Table:**

ROD, Synergy Custom system, HyPix-Arc 150 diffractometer	4404 independent reflections
Radiation source: Rotating-anode X-ray tube, Rigaku (Cu) X-ray Source	1823 reflections with *I* > 2σ(*I*)
Mirror monochromator	*R*_int_ = 0.051
Detector resolution: 10.0000 pixels mm^-1^	θ_max_ = 76.5°, θ_min_ = 3.4°
ω scans	*h* = −5→6
Absorption correction: multi-scan (CrysAlisPro; Rigaku OD, 2022)	*k* = −16→16
*T*_min_ = 0.889, *T*_max_ = 1.000	*l* = −21→20
11899 measured reflections	

### (±,E)-N'-(6-Methoxy-2-phenylchroman-4-ylidene)-2-(naphthalen-1-yloxy)-acetohydrazide (III) Refinement

**Table:**

Refinement on *F*^2^	Hydrogen site location: inferred from neighbouring sites
Least-squares matrix: full	H-atom parameters constrained
*R*[*F*^2^ > 2σ(*F*^2^)] = 0.077	*w* = 1/[σ^2^(*F*_o_^2^) + (0.1489*P*)^2^] where *P* = (*F*_o_^2^ + 2*F*_c_^2^)/3
*wR*(*F*^2^) = 0.285	(Δ/σ)_max_ < 0.001
*S* = 0.99	Δρ_max_ = 0.31 e Å^−^^3^
4404 reflections	Δρ_min_ = −0.24 e Å^−^^3^
309 parameters	Extinction correction: SHELXL-2018/3 (Sheldrick 2015), Fc^*^=kFc[1+0.001xFc^2^λ^3^/sin(2θ)]^-1/4^
6 restraints	Extinction coefficient: 0.0075 (16)

### (±,E)-N'-(6-Methoxy-2-phenylchroman-4-ylidene)-2-(naphthalen-1-yloxy)-acetohydrazide (III) Special details

**Table:**

Geometry. All esds (except the esd in the dihedral angle between two l.s. planes) are estimated using the full covariance matrix. The cell esds are taken into account individually in the estimation of esds in distances, angles and torsion angles; correlations between esds in cell parameters are only used when they are defined by crystal symmetry. An approximate (isotropic) treatment of cell esds is used for estimating esds involving l.s. planes.

### (±,E)-N'-(6-Methoxy-2-phenylchroman-4-ylidene)-2-(naphthalen-1-yloxy)-acetohydrazide (III) Fractional atomic coordinates and isotropic or equivalent isotropic displacement parameters (Å^2^)

**Table:**

	*x*	*y*	*z*	*U*_iso_*/*U*_eq_	
O1	0.1662 (5)	0.3408 (2)	0.51160 (17)	0.0808 (9)	
O2	0.3870 (6)	0.5290 (2)	0.18760 (17)	0.0873 (10)	
O3	1.2146 (6)	0.0164 (2)	0.41472 (17)	0.0801 (9)	
O4	0.9806 (6)	0.1395 (2)	0.24739 (15)	0.0734 (9)	
N1	0.7481 (6)	0.2143 (2)	0.38761 (19)	0.0665 (9)	
N2	0.8829 (6)	0.1260 (3)	0.42458 (19)	0.0676 (9)	
H2	0.836029	0.091524	0.473277	0.081*	
C1	0.5738 (8)	0.2504 (3)	0.4307 (2)	0.0640 (10)	
C9	0.4980 (9)	0.2062 (3)	0.5185 (2)	0.0783 (13)	
H9A	0.388021	0.146606	0.524494	0.094*	
H9B	0.656544	0.183096	0.547337	0.094*	
C8	0.3562 (11)	0.2788 (4)	0.5551 (3)	0.1022 (18)	
H8	0.493593	0.327843	0.556598	0.123*	
C2	0.4336 (7)	0.3430 (3)	0.3891 (2)	0.0628 (10)	
C7	0.2328 (8)	0.3834 (3)	0.4300 (2)	0.0690 (11)	
C6	0.0890 (8)	0.4682 (3)	0.3908 (3)	0.0789 (13)	
H6	−0.046360	0.494164	0.419216	0.095*	
C5	0.1448 (9)	0.5139 (4)	0.3106 (3)	0.0803 (13)	
H5	0.046166	0.570841	0.284228	0.096*	
C4	0.3478 (8)	0.4764 (3)	0.2679 (3)	0.0709 (12)	
C3	0.4898 (8)	0.3925 (3)	0.3065 (2)	0.0669 (11)	
H3	0.626100	0.367444	0.277822	0.080*	
C10	0.2606 (8)	0.2418 (3)	0.6417 (3)	0.0769 (13)	
C11	0.0709 (9)	0.1683 (4)	0.6671 (3)	0.0991 (16)	
H11	0.002437	0.138749	0.629577	0.119*	
C12	−0.0191 (11)	0.1377 (5)	0.7459 (4)	0.123 (2)	
H12	−0.148713	0.087663	0.761742	0.148*	
C13	0.0766 (14)	0.1789 (5)	0.8018 (4)	0.126 (3)	
H13	0.010054	0.158714	0.855512	0.151*	
C14	0.2700 (14)	0.2496 (5)	0.7791 (4)	0.122 (2)	
H14	0.340262	0.276882	0.817627	0.146*	
C15	0.3630 (10)	0.2812 (4)	0.6993 (3)	0.0975 (16)	
H15	0.496450	0.329700	0.684040	0.117*	
C16	1.0878 (8)	0.0944 (3)	0.3838 (2)	0.0635 (10)	
C17	1.1610 (8)	0.1602 (3)	0.2998 (2)	0.0686 (11)	
H17A	1.152062	0.232409	0.298819	0.082*	
H17B	1.339762	0.144127	0.282862	0.082*	
C18	0.9661 (8)	0.2057 (3)	0.1711 (2)	0.0664 (11)	
C19	1.1124 (9)	0.2940 (3)	0.1450 (2)	0.0783 (13)	
H19	1.228505	0.311241	0.179050	0.094*	
C20	1.0843 (10)	0.3574 (4)	0.0665 (3)	0.0871 (15)	
H20	1.181766	0.417527	0.049067	0.105*	
C21	0.9201 (9)	0.3338 (4)	0.0157 (3)	0.0835 (14)	
H21	0.906878	0.377231	−0.036360	0.100*	
C22	0.7663 (8)	0.2428 (4)	0.0409 (3)	0.0745 (12)	
C23	0.5937 (10)	0.2149 (4)	−0.0094 (3)	0.0892 (14)	
H23	0.577497	0.256913	−0.061720	0.107*	
C24	0.4485 (11)	0.1279 (5)	0.0160 (4)	0.1064 (17)	
H24	0.332690	0.110928	−0.018255	0.128*	
C25	0.4757 (11)	0.0636 (4)	0.0951 (3)	0.0992 (17)	
H25	0.378521	0.003345	0.112441	0.119*	
C26	0.6395 (8)	0.0876 (3)	0.1459 (3)	0.0782 (13)	
H26	0.653446	0.044377	0.197940	0.094*	
C27	0.7907 (8)	0.1786 (3)	0.1205 (2)	0.0665 (11)	
C28	0.6094 (9)	0.4996 (4)	0.1449 (3)	0.0982 (17)	
H28A	0.624427	0.544273	0.090701	0.147*	
H28B	0.589265	0.429952	0.142465	0.147*	
H28C	0.765986	0.504397	0.172318	0.147*	

### (±,E)-N'-(6-Methoxy-2-phenylchroman-4-ylidene)-2-(naphthalen-1-yloxy)-acetohydrazide (III) Atomic displacement parameters (Å^2^)

**Table:**

	*U*^11^	*U*^22^	*U*^33^	*U*^12^	*U*^13^	*U*^23^
O1	0.0821 (18)	0.091 (2)	0.0612 (18)	0.0282 (16)	−0.0048 (15)	−0.0090 (15)
O2	0.092 (2)	0.084 (2)	0.0690 (19)	0.0149 (17)	−0.0009 (17)	0.0044 (16)
O3	0.0884 (19)	0.080 (2)	0.0646 (18)	0.0234 (16)	−0.0024 (15)	−0.0105 (15)
O4	0.0956 (19)	0.0717 (18)	0.0488 (15)	−0.0066 (15)	−0.0098 (14)	−0.0071 (13)
N1	0.0739 (19)	0.060 (2)	0.062 (2)	0.0149 (16)	−0.0072 (17)	−0.0109 (16)
N2	0.075 (2)	0.071 (2)	0.0536 (18)	0.0181 (17)	−0.0033 (16)	−0.0133 (16)
C1	0.068 (2)	0.062 (2)	0.061 (2)	0.0055 (19)	−0.006 (2)	−0.014 (2)
C9	0.084 (3)	0.086 (3)	0.061 (3)	0.018 (2)	−0.002 (2)	−0.016 (2)
C8	0.122 (4)	0.102 (4)	0.068 (3)	0.043 (3)	0.007 (3)	−0.004 (3)
C2	0.061 (2)	0.062 (2)	0.065 (2)	0.0057 (19)	−0.0061 (19)	−0.016 (2)
C7	0.070 (2)	0.075 (3)	0.059 (2)	0.005 (2)	−0.005 (2)	−0.014 (2)
C6	0.079 (3)	0.081 (3)	0.073 (3)	0.026 (2)	−0.006 (2)	−0.016 (2)
C5	0.082 (3)	0.075 (3)	0.076 (3)	0.018 (2)	−0.002 (2)	−0.010 (2)
C4	0.075 (2)	0.069 (3)	0.063 (3)	0.002 (2)	−0.008 (2)	−0.007 (2)
C3	0.070 (2)	0.067 (3)	0.060 (2)	0.005 (2)	−0.003 (2)	−0.011 (2)
C10	0.081 (3)	0.078 (3)	0.066 (3)	0.017 (2)	0.004 (2)	−0.014 (2)
C11	0.098 (3)	0.116 (4)	0.085 (4)	−0.008 (3)	−0.010 (3)	−0.028 (3)
C12	0.108 (4)	0.142 (6)	0.101 (5)	−0.007 (4)	0.019 (4)	−0.005 (4)
C13	0.155 (6)	0.137 (6)	0.070 (4)	0.055 (5)	0.005 (4)	−0.010 (4)
C14	0.165 (6)	0.125 (5)	0.090 (5)	0.039 (5)	−0.047 (4)	−0.050 (4)
C15	0.104 (4)	0.089 (4)	0.101 (4)	0.001 (3)	−0.020 (3)	−0.025 (3)
C16	0.068 (2)	0.067 (3)	0.055 (2)	0.009 (2)	−0.0058 (19)	−0.016 (2)
C17	0.078 (2)	0.077 (3)	0.049 (2)	0.000 (2)	−0.006 (2)	−0.013 (2)
C18	0.079 (2)	0.066 (3)	0.048 (2)	0.006 (2)	0.000 (2)	−0.0062 (19)
C19	0.094 (3)	0.073 (3)	0.060 (3)	−0.007 (2)	−0.006 (2)	−0.004 (2)
C20	0.102 (3)	0.079 (3)	0.069 (3)	−0.007 (3)	0.000 (3)	0.000 (2)
C21	0.095 (3)	0.085 (3)	0.062 (3)	0.003 (3)	−0.002 (3)	−0.005 (2)
C22	0.080 (3)	0.081 (3)	0.061 (3)	0.018 (2)	−0.009 (2)	−0.018 (2)
C23	0.099 (3)	0.104 (4)	0.066 (3)	0.019 (3)	−0.025 (3)	−0.022 (3)
C24	0.110 (4)	0.121 (5)	0.098 (4)	0.007 (4)	−0.032 (3)	−0.039 (4)
C25	0.105 (4)	0.103 (4)	0.093 (4)	−0.016 (3)	−0.019 (3)	−0.027 (3)
C26	0.086 (3)	0.079 (3)	0.069 (3)	0.000 (2)	−0.007 (2)	−0.018 (2)
C27	0.072 (2)	0.073 (3)	0.055 (2)	0.008 (2)	−0.005 (2)	−0.017 (2)
C28	0.096 (3)	0.102 (4)	0.082 (3)	0.002 (3)	0.010 (3)	−0.004 (3)

### (±,E)-N'-(6-Methoxy-2-phenylchroman-4-ylidene)-2-(naphthalen-1-yloxy)-acetohydrazide (III) Geometric parameters (Å, º)

**Table:**

O1—C8	1.376 (5)	C12—H12	0.9300
O1—C7	1.383 (4)	C12—C13	1.349 (7)
O2—C4	1.371 (5)	C13—H13	0.9300
O2—C28	1.411 (5)	C13—C14	1.351 (6)
O3—C16	1.228 (5)	C14—H14	0.9300
O4—C17	1.404 (5)	C14—C15	1.374 (6)
O4—C18	1.374 (4)	C15—H15	0.9300
N1—N2	1.376 (4)	C16—C17	1.503 (5)
N1—C1	1.284 (5)	C17—H17A	0.9700
N2—H2	0.8600	C17—H17B	0.9700
N2—C16	1.338 (5)	C18—C19	1.374 (5)
C1—C9	1.488 (5)	C18—C27	1.401 (6)
C1—C2	1.453 (5)	C19—H19	0.9300
C9—H9A	0.9700	C19—C20	1.399 (6)
C9—H9B	0.9700	C20—H20	0.9300
C9—C8	1.451 (6)	C20—C21	1.345 (7)
C8—H8	0.9800	C21—H21	0.9300
C8—C10	1.485 (6)	C21—C22	1.425 (6)
C2—C7	1.382 (5)	C22—C23	1.392 (6)
C2—C3	1.406 (5)	C22—C27	1.414 (6)
C7—C6	1.376 (6)	C23—H23	0.9300
C6—H6	0.9300	C23—C24	1.357 (7)
C6—C5	1.360 (5)	C24—H24	0.9300
C5—H5	0.9300	C24—C25	1.410 (7)
C5—C4	1.387 (6)	C25—H25	0.9300
C4—C3	1.361 (5)	C25—C26	1.345 (6)
C3—H3	0.9300	C26—H26	0.9300
C10—C11	1.366 (5)	C26—C27	1.416 (6)
C10—C15	1.372 (6)	C28—H28A	0.9600
C11—H11	0.9300	C28—H28B	0.9600
C11—C12	1.352 (6)	C28—H28C	0.9600
			
C8—O1—C7	115.8 (3)	C13—C14—H14	120.0
C4—O2—C28	116.6 (4)	C13—C14—C15	120.1 (6)
C18—O4—C17	118.8 (3)	C15—C14—H14	120.0
C1—N1—N2	118.2 (3)	C10—C15—C14	120.6 (5)
N1—N2—H2	120.4	C10—C15—H15	119.7
C16—N2—N1	119.1 (3)	C14—C15—H15	119.7
C16—N2—H2	120.4	O3—C16—N2	121.2 (3)
N1—C1—C9	126.7 (4)	O3—C16—C17	121.6 (3)
N1—C1—C2	116.4 (3)	N2—C16—C17	117.2 (4)
C2—C1—C9	116.8 (3)	O4—C17—C16	107.4 (3)
C1—C9—H9A	109.0	O4—C17—H17A	110.2
C1—C9—H9B	109.0	O4—C17—H17B	110.2
H9A—C9—H9B	107.8	C16—C17—H17A	110.2
C8—C9—C1	113.0 (4)	C16—C17—H17B	110.2
C8—C9—H9A	109.0	H17A—C17—H17B	108.5
C8—C9—H9B	109.0	O4—C18—C27	116.1 (3)
O1—C8—C9	118.2 (5)	C19—C18—O4	122.8 (4)
O1—C8—H8	102.8	C19—C18—C27	121.1 (4)
O1—C8—C10	109.7 (4)	C18—C19—H19	120.4
C9—C8—H8	102.8	C18—C19—C20	119.1 (5)
C9—C8—C10	117.8 (4)	C20—C19—H19	120.4
C10—C8—H8	102.8	C19—C20—H20	119.1
C7—C2—C1	120.0 (4)	C21—C20—C19	121.8 (4)
C7—C2—C3	117.8 (4)	C21—C20—H20	119.1
C3—C2—C1	122.1 (4)	C20—C21—H21	119.8
C2—C7—O1	121.9 (4)	C20—C21—C22	120.4 (4)
C6—C7—O1	117.0 (4)	C22—C21—H21	119.8
C6—C7—C2	121.1 (4)	C23—C22—C21	122.6 (4)
C7—C6—H6	120.0	C23—C22—C27	119.0 (4)
C5—C6—C7	120.0 (4)	C27—C22—C21	118.4 (4)
C5—C6—H6	120.0	C22—C23—H23	119.2
C6—C5—H5	119.7	C24—C23—C22	121.7 (5)
C6—C5—C4	120.5 (4)	C24—C23—H23	119.2
C4—C5—H5	119.7	C23—C24—H24	120.4
O2—C4—C5	115.4 (4)	C23—C24—C25	119.1 (5)
C3—C4—O2	125.1 (4)	C25—C24—H24	120.4
C3—C4—C5	119.6 (4)	C24—C25—H25	119.4
C2—C3—H3	119.5	C26—C25—C24	121.3 (5)
C4—C3—C2	121.0 (4)	C26—C25—H25	119.4
C4—C3—H3	119.5	C25—C26—H26	119.9
C11—C10—C8	122.0 (5)	C25—C26—C27	120.3 (4)
C11—C10—C15	117.8 (4)	C27—C26—H26	119.9
C15—C10—C8	120.2 (5)	C18—C27—C22	119.3 (4)
C10—C11—H11	119.5	C18—C27—C26	122.0 (4)
C12—C11—C10	121.0 (5)	C22—C27—C26	118.7 (4)
C12—C11—H11	119.5	O2—C28—H28A	109.5
C11—C12—H12	119.6	O2—C28—H28B	109.5
C13—C12—C11	120.9 (6)	O2—C28—H28C	109.5
C13—C12—H12	119.6	H28A—C28—H28B	109.5
C12—C13—H13	120.2	H28A—C28—H28C	109.5
C12—C13—C14	119.5 (6)	H28B—C28—H28C	109.5
C14—C13—H13	120.2		
			
O1—C8—C10—C11	73.5 (6)	C7—C6—C5—C4	−0.6 (7)
O1—C8—C10—C15	−106.7 (6)	C6—C5—C4—O2	−179.6 (4)
O1—C7—C6—C5	179.8 (4)	C6—C5—C4—C3	0.7 (7)
O2—C4—C3—C2	−179.5 (4)	C5—C4—C3—C2	0.1 (6)
O3—C16—C17—O4	103.6 (5)	C3—C2—C7—O1	−179.0 (4)
O4—C18—C19—C20	−179.6 (4)	C3—C2—C7—C6	1.2 (6)
O4—C18—C27—C22	−179.7 (4)	C10—C11—C12—C13	−0.2 (10)
O4—C18—C27—C26	−0.7 (6)	C11—C10—C15—C14	−2.1 (8)
N1—N2—C16—O3	179.5 (4)	C11—C12—C13—C14	−1.8 (11)
N1—N2—C16—C17	1.4 (5)	C12—C13—C14—C15	1.7 (10)
N1—C1—C9—C8	162.8 (5)	C13—C14—C15—C10	0.2 (9)
N1—C1—C2—C7	176.4 (4)	C15—C10—C11—C12	2.1 (8)
N1—C1—C2—C3	−1.7 (6)	C17—O4—C18—C19	−2.9 (6)
N2—N1—C1—C9	−0.2 (6)	C17—O4—C18—C27	177.3 (3)
N2—N1—C1—C2	−179.2 (3)	C18—O4—C17—C16	165.4 (3)
N2—C16—C17—O4	−78.3 (4)	C18—C19—C20—C21	−0.7 (8)
C1—N1—N2—C16	−171.6 (4)	C19—C18—C27—C22	0.5 (6)
C1—C9—C8—O1	42.0 (7)	C19—C18—C27—C26	179.5 (4)
C1—C9—C8—C10	177.5 (4)	C19—C20—C21—C22	0.6 (8)
C1—C2—C7—O1	2.8 (6)	C20—C21—C22—C23	−180.0 (5)
C1—C2—C7—C6	−176.9 (4)	C20—C21—C22—C27	0.1 (7)
C1—C2—C3—C4	177.0 (4)	C21—C22—C23—C24	−179.8 (5)
C9—C1—C2—C7	−2.7 (6)	C21—C22—C27—C18	−0.7 (6)
C9—C1—C2—C3	179.2 (4)	C21—C22—C27—C26	−179.7 (4)
C9—C8—C10—C11	−65.5 (7)	C22—C23—C24—C25	−0.8 (8)
C9—C8—C10—C15	114.3 (6)	C23—C22—C27—C18	179.4 (4)
C8—O1—C7—C2	19.7 (6)	C23—C22—C27—C26	0.4 (6)
C8—O1—C7—C6	−160.6 (5)	C23—C24—C25—C26	1.0 (9)
C8—C10—C11—C12	−178.1 (5)	C24—C25—C26—C27	−0.5 (8)
C8—C10—C15—C14	178.1 (5)	C25—C26—C27—C18	−179.2 (4)
C2—C1—C9—C8	−18.2 (6)	C25—C26—C27—C22	−0.2 (7)
C2—C7—C6—C5	−0.4 (7)	C27—C18—C19—C20	0.2 (7)
C7—O1—C8—C9	−43.1 (6)	C27—C22—C23—C24	0.1 (7)
C7—O1—C8—C10	178.1 (4)	C28—O2—C4—C5	173.4 (4)
C7—C2—C3—C4	−1.1 (6)	C28—O2—C4—C3	−6.9 (6)

### (±,E)-N'-(6-Methoxy-2-phenylchroman-4-ylidene)-2-(naphthalen-1-yloxy)-acetohydrazide (III) Hydrogen-bond geometry (Å, º)

**Table:**

*D*—H···*A*	*D*—H	H···*A*	*D*···*A*	*D*—H···*A*
N2—H2···O3^i^	0.86	2.08	2.922 (4)	167

Symmetry code: (i) −*x*+2, −*y*, −*z*+1.
